# A *Clostridium difficile* Cell Wall Glycopolymer Locus Influences Bacterial Shape, Polysaccharide Production and Virulence

**DOI:** 10.1371/journal.ppat.1005946

**Published:** 2016-10-14

**Authors:** Michele Chu, Michael J. G. Mallozzi, Bryan P. Roxas, Lisa Bertolo, Mario A. Monteiro, Al Agellon, V. K. Viswanathan, Gayatri Vedantam

**Affiliations:** 1 School of Animal and Comparative Biomedical Sciences, University of Arizona, Tucson, Arizona, United States of America; 2 Department of Chemistry, University of Guelph, Guelph, Ontario, Canada; 3 Department of Immunobiology, Bio5 Institute for Collaborative Research, University of Arizona, Tucson, Arizona, United States of America; 4 Southern Arizona VA Healthcare System, Tucson, Arizona, United States of America; University of Texas Medical School at Houston, UNITED STATES

## Abstract

*Clostridium difficile* is a diarrheagenic pathogen associated with significant mortality and morbidity. While its glucosylating toxins are primary virulence determinants, there is increasing appreciation of important roles for non-toxin factors in *C*. *difficile* pathogenesis. Cell wall glycopolymers (CWGs) influence the virulence of various pathogens. Five *C*. *difficile* CWGs, including PSII, have been structurally characterized, but their biosynthesis and significance in *C*. *difficile* infection is unknown. We explored the contribution of a conserved CWG locus to *C*. *difficile* cell-surface integrity and virulence. Attempts at disrupting multiple genes in the locus, including one encoding a predicted CWG exporter *mviN*, were unsuccessful, suggesting essentiality of the respective gene products. However, antisense RNA-mediated *mviN* downregulation resulted in slight morphology defects, retarded growth, and decreased surface PSII deposition. Two other genes, *lcpA* and *lcpB*, with putative roles in CWG anchoring, could be disrupted by insertional inactivation. *lcpA* ^-^ and *lcpB* ^-^ mutants had distinct phenotypes, implying non-redundant roles for the respective proteins. The *lcpB* ^-^ mutant was defective in surface PSII deposition and shedding, and exhibited a remodeled cell surface characterized by elongated and helical morphology, aberrantly-localized cell septae, and an altered surface-anchored protein profile. Both *lcpA* ^-^ and *lcpB* ^-^ strains also displayed heightened virulence in a hamster model of *C*. *difficile* disease. We propose that gene products of the *C*. *difficile* CWG locus are essential, that they direct the production/assembly of key antigenic surface polysaccharides, and thereby have complex roles in virulence.

## Author Summary

*Clostridium difficile* infection is a leading healthcare-onset bacterial disease, and its management and prevention imposes significant clinical and financial burdens worldwide. While toxins TcdA and TcdB are the primary virulence factors, there is increasing interest in, and appreciation of, non-toxin virulence factors in *C*. *difficile* pathogenesis. Cell wall glycopolymers (CWGs) are important virulence determinants in many pathogens, but their role(s) in *C*. *difficile* pathogenesis is unclear. We propose a model for *C*. *difficile* CWG biosynthesis, and demonstrate that alterations in cell wall assembly profoundly impact bacterial morphology and virulence.

## Introduction

*Clostridium difficile* is a Gram-positive, anaerobic, spore-forming, enteric pathogen. It is a causative agent of antibiotic-associated diarrhea and, in a subset of patients, can engender severe sequelae including pseudomembranous colitis [1,2]. *C*. *difficile* infection (CDI) severely impacts healthcare systems across North America [3,4], and the pathogen has been designated an “Urgent Threat” by the Centers for Disease Control and Prevention [5]. There are over 500,000 CDI cases per year in the U.S.A, with an associated annual economic burden of approximately $4 billion in excess healthcare costs [4,6].

Toxins A and B (TcdA and TcdB respectively), the primary *C*. *difficile* virulence factors, are significant mediators of intestinal damage and pathology [7–9]. However, the contributions of non-toxin virulence factors to *C*. *difficile* colonization and disease is becoming increasingly appreciated [10]. Cell wall glycopolymers (CWGs), including capsular polysaccharides and teichoic acids, are known virulence factors in other pathogenic bacteria [11,12]. CWGs have diverse roles, including protection against desiccation, host-cell adhesion, resistance to antimicrobial agents, and immune evasion [12]. Davies and Borriello showed the presence of a capsule-like layer in diverse *C*. *difficile* strains over twenty years ago, and noted that there was no obvious correlation between presence of a capsule and virulence [13]. Contrary to these observation, Baldessarri et al. found that strains from symptomatic patients exhibited a thicker CWG layer [14]. The actual contribution of *C*. *difficile* CWGs to colonization and pathogenesis, however, has not been explored.

Several *C*. *difficile* surface polysaccharides, including the water-soluble PSI and PSII, and phenol-soluble PSIII (a lipoteichoic acid), have been structurally characterized [15,16]. PSI is expressed at low levels on the cell surface, and it is unclear if it is present across diverse *C*. *difficile* strains; PSII and PSIII, however, appear to be conserved surface polysaccharide antigens amongst all *C*. *difficile* strains tested [15–20]. Thus far, the biological significance of PSI, PSII and PSIII in virulence is unknown. Willing et al. recently identified a genetic locus that is likely involved in CWG biosynthesis [21]. The proteins encoded by this cluster include a putative initiating transferase (CD2783), flippase-type exporter (MviN), polymerase (CD2777), and LytR-CpsA-Psr (LCP)-like proteins (LcpA and LcpB); this locus is reminiscent of the *Streptococcus pneumoniae* capsule-synthesis cassette [22,23].

In this study, we used multiple approaches to demonstrate the presence of a CWG layer in *C*. *difficile*, and developed an immunoblot-based assay to specifically detect PSII. Further, we explored the roles of several genes in the CWG locus, specifically *mviN*, *lcpA* and *lcpB*, in PSII- and total polysaccharide-production, and virulence. Collectively, our data extend those presented in previous studies, and strongly support a role for gene products of the *C*. *difficile* CWG locus in surface polysaccharide anchoring and *C*. *difficile* virulence, and suggest that alteration or abrogation of their expression (where tolerated) induces pleotropic changes.

## Materials and Methods

### Ethics statement

Studies were approved by the University of Arizona Institutional Animal Care and Use Committee (IACUC) and under a permit issued to the Vedantam laboratory.

### Bacterial strains and propagation

The strains used in this study are listed in Table 1. Strains *C*. *difficile* 630 and *Clostridium perfringens* (GV339) were a kind gift from Dr. Dale Gerding, Hines VA Hospital, Hines, IL. *Clostridium botulinum* (GV340), was a kind gift from the Microbiology Teaching Laboratory Collection at the University of Arizona. JIR8094 [24] was a kind gift from Dr. Bruno Dupuy, Pasteur Institute, France. Unless otherwise indicated, ribotype information is provided to denote molecular type of *C*. *difficile* [25]. *C*. *difficile* was propagated in un-supplemented brain heart infusion (BHI) or BHI supplemented with 0.5% yeast extract and 0.1% cysteine (BHIS). TY medium (2% tryptone and 3% yeast extract) was used for antisense-RNA induction studies. Unless otherwise stated, all antibiotics (including susceptibility testing disks) were purchased from Sigma-Aldrich (St. Louis, MO) and used at the concentrations indicated.

**Table 1:** Strains, plasmids and primers used in this study.

Strains, plasmids, primer name		Genotype and/or phenotype, primer sequence	Source or reference
*C*. *difficile*			
	630	Wild type, ribotype 012	35, 42
	GV1	Wild type, ribotype 078	42
	GV41	Wild type, ribotype 003	42
	GV44	Wild type, ribotype 027, REA type BI-1	Dale Gerding, Hines VA
	GV45	Wild type, ribotype 027, REA type BI-6	Dale Gerding, Hines VA
	GV46	Wild type, ribotype 027, hypervirulent, REA type BI-8	42
	GV48	Wild type, ribotype 027, REA type BI-17	Dale Gerding, Hines VA
	GV51	Wild type, ribotype 027, REA type BI-23	Dale Gerding, Hines VA
	GV53	Wild type, ribotype 017, hypervirulent	Dale Gerding, Hines VA
	GV59	Wild type, ribotype 001	42
	GV60	Wild type, ribotype 053	42
	GV64	Wild type, ribotype 106	Dale Gerding, Hines VA
	GV66	Wild type, ribotype 009, non-toxigenic	Dale Gerding, Hines VA
	GV71	Wild type, ribotype 010, non-toxigenic	ATCC
	GV83	Wild type, ribotype 015	Glenn Songer, Iowa State University
	GV85	Wild type, ribotype 020	Glenn Songer, Iowa State University
	JIR8094 (WT)	Erm^S^ strain of 630	24
	GV341 (*mviN* KD)	JIR8094, bears pMC6; contains inducible *mviN* asRNA fragment	This study
	GV342	JIR8094, bears pRPF185	This study
	GV343 (*lcpA* ^-^)	JIR8094, ClosTron insertion in *lcpA*	This study
	GV344 (*lcpB* ^-^)	JIR8094, ClosTron insertion in *lcpB*	This study
	GV345 (*lcpA* ^-^ p*lcpA*)	JIR8094, ClosTron insertion in *lcpA* bearing pMC9; *lcpA* complement strain	This study
	GV346 (*lcpB* ^-^ p*lcpB*)	JIR8094, ClosTron insertion in *lcpB* bearing pMC10; *lcpB* complement strain	This study
	GV347 [*lcpA* ^-^ (Vector)]	JIR8094, ClosTron insertion in *lcpA* bearing pMAC1; *lcpA* vector control	This study
	GV348 [*lcpB* ^-^ (Vector)]	JIR8094, ClosTron insertion in *lcpB* bearing pMAC1; *lcpB* vector control	This study
	GV349 [WT (Vector)]	JIR8094, bears pMAC1; WT vector control	This study
	GV435	GV44, ClosTron insertion in *lcpB*	This study
	GV436	GV66, ClosTron insertion in *lcpB*	This study
*C*. *perfringens*			
	GV339	Unknown	Dale Gerding, Hines VA
*C*. *botulinum*			
	GV340	Unknown	University of Arizona Microbiology Teaching Laboratory
*E*. *coli*			
	CA434	HB101 harboring the broad host-range plasmid R702	31
Plasmids			
	pRPF185	Inducible expression vector (contains *gusA* under the control of P_tet_)	29
	pMTL007C-E5	Parent plasmid used to generate specific ClosTron insertions in multiple *C*. *difficile* genes	26
	pMTL82153	Constitutive expression vector	28
	pMC4	pMTL82153 bearing a 107bp *mviN* asRNA fragment	Synthesized by DNA 2.0
	pMC6	pRPF185 bearing a 107bp *mviN* asRNA fragment	This study
	pMC9	pRPF185 bearing a WT copy of *lcpA* (p*lcpA*)	This study
	pMC10	pRPF185 bearing a WT copy of *lcpB* (p*lcpB*)	This study
	pMAC1	pRPF185 lacking *gusA* (Empty vector)	This study
Primers			
	MGM119	5’-GATCGAGCTCTATGTGCAAGTACAAGTTCTCTAAGGA-3’	This study
	MGM120	5’-GATCGGATCCTGCCTTAGAATCCATTACAGACTTATCC-3’	This study
	MC25	5’-GCGTTAACAGATCTGTTGTCAAAATTAAAGAAATTTGTT-3’	This study
	MC26	5’-AAAACTTATAGGATCTTATTGTTTAAACTCTATGTCAT-3’	This study
	MC29	5’-GCGTTAACAGATCTGTTGTCAGGACTCAAAAAGTTC-3’	This study
	MC30	5’-AAAACTTATAGGATCCTAATCTTCAACCATAATATCT-3’	This study
	MC46	5’- TTATGGGTTCAGGAACAATTAAAGAA-3’	This study
	MC47	5’- TTAAAACGCCCAGTGTCGC-3’	This study
	MC48	5’- TGGATAGCTATAAGACCTTCAGGTACA-3’	This study
	MC49	5’- ATAGTTTCGTTTATTAAAGCATCTTCT-3’	This study
	MC50	5’-AAATGATGCAGTTATGGTTTGTAGAA-3’	This study
	MC51	5’-TATCTCATATTCTGGAAGCTTTTCTTT-3’	This study
	MC52	5’-GTAATGCAGGCTATGTAATACCTTTTG-3’	This study
	MC53	5’-AAAATATCCATAGACACTTATAGGGAA-3’	This study

### Plasmids and mutagenesis

A list of the plasmids and primers used in this study is listed in Table 1. *C*. *difficile* gene-specific disruptions were designed using the intron-targeting tool ClosTron [26]. Seven open reading frames (ORFs) from the putative CWG locus, based on the *C*. *difficile* strain 630 genome sequence, were targeted for disruption: CD2783, CD2781 (*mviN*), CD2780 (*pgm2*), CD2779 (*manC*), CD2769, CD2765 (*lcpA*), and CD2766 (*lcpB*). The gene-specific ClosTron insertion site was chosen based on the Perutka algorithm [27]. Targeting regions were constructed and cloned into pMTL007C-E5 by DNA 2.0 (Menlo Park, CA; [26]). Knockdown of *mviN* expression was achieved via the inducible expression of a 170 base pair RNA fragment complementary to the ribosome-binding site and the first 30 codon-encoding nucleotides of the *mviN* mRNA. This DNA fragment was synthesized and cloned into pMTL82153 [28] by DNA2.0 (Menlo Park, CA) to generate pMC4. To facilitate inducible expression, the insert was amplified using primers MGM119 and MGM120 (Table 1), digested with *Sac*I and *Bam*HI restriction enzymes (New England Biolabs, Ipswich, MA), and cloned into similarly digested pRPF185 (kind gift from Drs. Neil Fairweather and Robert Fagan) to generate pMC6 [29]. The *lcpA-* and *lcpB-*complementing plasmids were derived via PCR amplification from JIR8094 chromosomal DNA using the corresponding primers (MC29/MC30, and MC25/MC26, respectively; Table 1). The In-Fusion HD Cloning Kit (Clontech Laboratories, Inc., Mountain View, CA) was used to clone the fragments into pRPF185 to generate pMC9 (for *lcpA*) and pMC10 (for *lcpB*). For a corresponding control, pRPF185 was digested with *Sac*I and *Bam*HI, Klenow Polymerase-treated, and re-ligated using the T4 DNA ligase kit (Thermo-Fisher Scientific Inc., Waltham, MA) to yield pMAC1.

### *C*. *difficile* conjugation

Plasmids were transferred from *E*. *coli* to *C*. *difficile* via conjugation following the protocol of Heap et al. [30] with some modifications. ClosTron constructs for targeted *lcpA* and *lcpB* disruptions, respectively, were electroporated into *E*. *coli* CA434 [31]. *E*. *coli* CA434 containing one of the constructs of interest (donor strain) and *C*. *difficile* JIR8094 (recipient strain) were grown overnight in BHIS [24]. One milliliter (mL) of the overnight donor culture was centrifuged at 1,500 x g, washed in 1mL of BHIS, and pelleted again. The pellet was re-suspended in 200μL of the overnight recipient strain and plated on a BHIS plate incubated at 37°C anaerobically for 10 hours. The entire bacterial lawn was scraped off the plate, re-suspended in 1mL phosphate buffer saline (PBS), of which 200μL was plated on BHIS containing cefoxitin (8μg/mL), cycloserine (250μg/mL), and thiamphenicol (15μg/mL), and incubated anaerobically for 24–48 hours. *C*. *difficile* transconjugants were passaged on BHIS containing erythromycin (10μL/ml) to select for potential gene-specific integrants. Potential integrants were screened by PCR using gene-specific primers, paired with a primer specific for the antibiotic resistance cassette (ErmRAM) sequence [26].

### CWG visualization by transmission electron microscopy

*C*. *difficile* was visualized via thin-section electron microscopy using the pre- and post-fixation protocol of Davies and Boriello [13]. Lawns of *C*. *difficile* strains 630 and GV53 (K14) were grown on BHIS for 48 or 72 hours. For pre-fixation, cells were harvested from the plates in 5mL of fixative solution [0.1 M sodium cacodylate (Santa Cruz Biotech, Santa Cruz, CA), 10mM L-lysine, 0.1% ruthenium red, and 3% glutaraldehyde], and incubated at room temperature for 10 minutes. The fixative solution was removed and replaced with fresh fixative excluding L-lysine, and was incubated for two hours at room temperature. The cells were then washed three times in 0.1M sodium cacodylate buffer with 0.1% ruthenium red. For post-fixation, cells were suspended in 1% osmium tetroxide (Electron Microscopy Sciences, Hatfield, PA) in 0.1M sodium cacodylate buffer with 0.1% ruthenium red, and incubated at room temperature for two hours. Subsequently, the samples were subjected to an ethanol-water dehydration series consisting of 30%, 50%, 70%, 80%, and 90% ethanol for five minutes each, followed by 100% ethanol three times for 20 minutes each, or only 30%, 50%, then 70% ethanol three times for 20 minutes. The samples were then left in a 50:50 mixture of ethanol and the polyhydroxy aromatic acrylic resin LR White (Sigma-Aldrich, St. Louis, MO) overnight, followed by four washes in LR White resin, and incubation for an additional 24 hours at 50°C for polymerization. Blocks were sectioned and imaged at the AHSC Research Electron Microscopy facility (ARL Biotech, University of Arizona) using a FEI (Philips) CM12 transmission electron microscope.

Alternatively, GV346 (*lcpB* ^-^ p*lcpB*), GV348 [*lcpB* ^-^ (Vector)] and GV349 [WT (Vector)] were grown overnight in BHI broth, then harvested in PBS with calcium and magnesium and fixed with 1.5% paraformaldehyde and 0.2% glutaraldehyde. Samples were processed through the Imaging Cores of Arizona Research Labs (ARL), University of Arizona and imaged using the FEI Tecnai Spirit transmission electron microscope.

### CWG visualization by scanning electron microscopy

*C*. *difficile* capsular polysaccharides were visualized via scanning electron microscopy using the pre- and post-fixation protocols of Hammerschmidt et al. and Davies and Boriello [13,32]. Briefly, GV53 (*C*. *difficile* K14) was grown overnight in BHI broth, diluted 1:50, and grown to mid-log phase. The mid-log cultures were plated on BHI agar and grown for 24–36 hours. For pre-fixation, an agar plug was fixed in 0.1M sodium cacodylate buffer containing 0.1% ruthenium red, 3% glutaraldehyde, and 10mM L-lysine for 20 minutes on ice. The fixative solution was removed, and the plugs were washed with 0.1M sodium cacodylate buffer containing 0.1% ruthenium red. The plugs were fixed again in fixative solution without L-lysine for 3 hours at room temperature, washed in 0.1M sodium cacodylate buffer containing 0.1% ruthenium red, and then post-fixed in 0.1M sodium cacodylate buffer containing 0.1% ruthenium red and 1% osmium tetroxide for 1 hour at room temperature. Following post-fixation, the plugs were washed 3 times in 0.1M sodium cacodylate buffer containing 0.1% ruthenium red, subjected to an ethanol-water dehydration series consisting of 10%, 30%, 50%, 70%, 90%, and 100% at 10 minutes each on ice. At the final dehydration step, the plugs were warmed to room temperature and fresh 100% ethanol was added. Further processing and imaging of the plugs were done at the University of Arizona’s Spectroscopy and Imaging Facilities, and visualization performed using a Hitachi S-4800 Type II Ultra-High Resolution Field Emission Scanning Electron Microscope. Alternatively, strains GV345 (*lcpA* ^-^ p*lcpA*), GV346 (*lcpB* ^-^ p*lcpB*), GV347 [*lcpA* ^-^ (Vector)], GV348 [*lcpB* ^-^ (Vector)] and GV349 [WT (Vector)] were grown overnight in BHI broth, harvested and fixed in 2% glutaraldehyde and submitted to the University Spectroscopy and Imaging Facilities at the University of Arizona for processing and imaging using the same microscope as above.

### Bioinformatic analyses of the *C*. *difficile* CWG locus

The RAST (Rapid Annotation using Subsystem Technology) sequence-based comparative tool [33] was used to compute predicted proteomes of 32 *C*. *difficile* strains whose genome sequences were obtained from National Center for Biotechnology Information GenBank (NCBI; GenBank; [34]), as well as specific isolates in the Vedantam Laboratory strain collection, and compared to the *C*. *difficile* strain 630 genome [35] (S1 Table). The conditional formatting function of the Microsoft Excel application (Microsoft, Redmond, WA) was used to generate the protein identity/*C*. *difficile* strain grid. Different colors were assigned to varying ranges of percent identities in each cell. PHYLIP [36] was used to compute and generate a cladogram where strains with similar protein identity profiles were clustered together.

### Transcriptional analysis

For non-quantitative Reverse Transciptase (RT)-PCR analysis, JIR8094 was grown in BHI broth overnight then sub-cultured in BHI broth to a 600 nanometer (nm) optical density (OD)_600nm_ = 0.4. Cells were harvested by centrifugation, and RNA extracted using the GeneJET RNA Purification Kit (Thermo Fisher Scientific Inc., Waltham, MA). Extracted RNA was subsequently treated with DNAse (Thermo Fisher Scientific Inc., Waltham, MA) to remove any contaminating genomic DNA. Complementary DNA (cDNA) was synthesized from DNAse-treated RNA using the Superscript III First Strand kit (Thermo Fisher Scientific Inc., Waltham, MA). The intergenic regions of the following genes were amplified from the cDNA using conventional PCR, and the primers listed in Table 1: *mviN* and *pgm2* (MC46 and MC47), *pgm2* and *manC* (MC48 and MC49), *manC* and CD2778 (MC50 and MC51), and *lcpA* and *lcpB* (MC52 and MC53).

### Cell wall glycopolymer extraction and fractionation

Surface-extractable CWGs were isolated using a modified polysaccharide-extraction method previously described by Mueller et al. [37]. Briefly, *C*. *difficile* strains, GV1, GV41, GV44, GV45, GV46, GV48, GV51, GV53, GV59, GV60, GV64, GV71, GV83, GV85, GV339 and GV340 were grown in BHI broth overnight, standardized by (OD)_600nm_ readings, and washed once in PBS. Cells were pelleted by centrifugation for 10 minutes at 3220 x g. The wet cell pellet was re-suspended in ethylenediaminetetraacetic acid-triethylamine (EDTA-TEA) extraction buffer (50μL EDTA-TEA/20mg of wet cell mass) and incubated at room temperature for 15 minutes. The samples were then centrifuged at 10,000 x g for 2 minutes, and the supernatant was collected.

For cell wall fractionation and PSII shedding studies, strains were grown overnight, sub-cultured in 50mL BHI for 12 hours, harvested by centrifugation, and the supernatant (used for PSII harvesting) collected and concentrated through a 3kDa cutoff column to a final volume of 1.5mL. The remaining pellet (P1) was subjected to a total Surface-Layer Protein (SLP) shearing protocol as described by Fagan et al. [38]; this resulted in another supernatant (SLP fraction) and pellet (P2; finally used for cytosolic protein extraction). Briefly, for SLP shearing, the P1 pellet was washed in PBS, resuspended in 500μL of 0.2M glycine pH 2.2, and incubated at room temperature for 30 minutes with gentle agitation. Extracts were centrifuged at 16,000 x g for 15 minutes at 4°C and the supernatants were collected and neutralized with 2M tris-base. Finally, the remaining pellet (P2) was re-suspended in 50mM triethylammonium bicarbonate buffer, sonicated, and 6M urea was added to obtain a total cell lysate. For all subsequent immunoblot and SDS-PAGE analyses, all samples were normalized to total protein in the respective fraction (50μg for the supernatant fraction and 5μg for surface layer proteins; a representative Gel Code Blue-stained gel is presented in S6 Fig to show equivalent sample loading). Two independent biological replicates were performed for each strain. All SDS-PAGE analyses were performed using 4–20% TGX gels (Bio-Rad, Hercules, CA) followed by staining with Gel Code Blue (Thermo Fisher Scientific Inc., Waltham, MA).

### Immunoblot analyses

PSII conjugated to the heat-labile enterotoxin B subunit (LTB) of enterotoxigenic *Escherichia coli* (ETEC) was a kind gift from Dr. Mario Monteiro [18]. We submitted this conjugate (PSII-LTB) to Alpha Diagnostics (San Antonio, Texas) for polyclonal antibody generation in rabbits (intramuscular immunization with 1mg of the conjugate per animal). The pre-immune serum and two bleeds were collected at Days 1, 53 and 67, respectively. For PSII immunoblot analysis, crude extracts normalized to cell number or total protein were diluted two-fold in PBS, and 5μL spotted onto an activated polyvinylidene fluoride (PVDF) membrane. The membrane was vigorously washed in deionized water for 20 minutes, blocked in 10% milk in TBST (Tris-buffered saline with 0.1% tween 20) for 15 minutes, and then washed in 5% milk in TBST for 2 minutes. After overnight incubation in primary antibody (pre-immune rabbit serum or PSII-LTB rabbit antiserum, 1:8,000), the membrane was washed once in 5% milk in TBST, twice in TBST for 5 minutes each, and once in 5% milk in TBST for 10 minutes. Following incubation with goat anti-rabbit horseradish-peroxidase-conjugated secondary antibody (Sigma-Aldrich, St. Louis, MO) at 1:10,000 dilution for 1 hour, the membrane was washed 5 times in TBST for 5 minutes each, and developed using SuperSignal West Femto chemiluminescent substrate (Thermo Fisher Scientific Inc., Waltham, MA).

### Antisense RNA-mediated *mviN* down-regulation

*C*. *difficile* JIR8094 containing pMC6 or pRPF185 (GV341 and GV342 respectively) were grown overnight in TY broth supplemented with thiamphenicol (15μg/mL). The strains were sub-cultured (1:50 or 1:100) in TY broth with or without anhydrotetracycline (500ng/mL) and grown for 12 hours. Following the induction, the cells were imaged using the Maneval’s capsule stain as described by Corstvet et al. [39] and processed for PSII production using the EDTA-TEA extraction method and immunoblot analysis as described above.

### *C*. *difficile* growth and toxin assessments

*C*. *difficile* JIR8094 containing pMC6 or pRPF185 (GV341 and GV342 respectively), were grown overnight in TY broth supplemented with thiamphenicol as described above. The strains were sub-cultured (1:50), grown to an (OD)_600nm_ of approximately 0.6, and then diluted 1:100 in 30mL of TY broth with thiamphenicol and with or without 500ng/mL anhydrotetracycline. OD measurements were taken within a 24-hour time period. Two biological replicates were performed for each condition.

*C*. *difficile* JIR8094, and isogenic *lcpA* ^-^ and *lcpB* ^-^ mutants (GV343 and GV344 for the *lcpA* ^-^ and *lcpB* ^-^ mutants respectively) were grown overnight in BHI broth. The strains were standardized to (OD)_600nm_ = 0.1 and monitored over a 12-hour time period with (OD)_600nm_ measurements taken every hour. Three biological replicates were performed for each strain. Toxin data was measured at 72 hours from culture supernatants using the Wampole *C*. *difficile* Toxin A/B ELISA kit (Alere, Waltham MA, USA). Three biological replicates were used in the *C*. *difficile* toxin ELISA analysis.

### Proteomic analyses of *mviN* knock-down strains

Strains GV341 and GV342 were grown in TY broth with thiamphenicol (15 μg/mL) overnight, then diluted 1:50 in TY and thiamphenicol with or without anhydrotetracycline (500ng/mL). Samples were collected at mid-log and processed for iTRAQ proteomic analysis as described previously [40,41]. Briefly, a total of 100 μg of protein in 20 μl of 7M urea, 2M thiourea, and 4% CHAPS (Sigma-Aldrich, St. Louis, MO) was denatured in 2% SDS, reduced in 100mM tris-(2-carboxyethyl) phosphine, and alkylated in 84mM iodoacetamide. The urea concentration of the samples was diluted to 2M using 100mM TEAB prior to digestion with trypsin at a ratio of 1:10. iTRAQ reagent labeling was performed according to manufacturers’ instructions (AB SCIEX, Framingham, MA). Strong cationic exchange (SCX) fractionation was performed using Waters 600E HPLC system, fractions collected, resuspended in acetonitrile/ trifluoroacetic acid, injected onto a Merck Chromolith CapRod column, and eluates automatically spotted on a stainless steel MALDI target plate for spot analysis (ABSciexTripleTOF 5600+ mass spectrophotometer; AB SCIEX, Framingham, MA). Protein identification and quantitation was performed using the Protein Pilot 3.0 software against the *C*. *difficile* strain 630 protein database, differentially abundant proteins were identified via multiple stringent statistical tests as described previously [40,41], and in the Statistics section below.

### Immunofluorescence studies

To immobilize bacteria, 10μL of a 1:10 dilution of poly-L-lysine was spotted on a multi-well slide (MP Biomedicals, LLC., Santa Ana, CA) for 5 minutes, followed by 2 washes with PBS. Strains GV341 and GV342 were sub-cultured overnight in BHI broth with anhydrotetracycline (500ng/mL) then diluted to an (OD)_600nm_ = 0.1. Equal volumes were centrifuged at 3220 x g for 10 minutes, then re-suspended in 500μL of BHI. Ten microliters of the culture was spotted on poly-L-lysine coated slides, and fixed with 1.5% paraformaldehyde for 15 minutes at room temperature. The wells were washed 3 times with PBS for 5 minutes each, incubated in 50mM ammonium chloride for 5 minutes, washed 2 times with PBS for 5 minutes each, then blocked for 30 minutes with 2% IgG-free bovine serum albumin (BSA) in PBS. Cells were incubated in PSII-LTB rabbit antiserum (at a 1:100 dilution) for one hour, washed 5 times with 1% BSA for 5 minutes each, incubated with Alexafluor 488 at a 1:500 dilution (Life Technologies, Grand Island, NY) for one hour, followed by 6 washes in 1% BSA and 2 washes in PBS. Samples were mounted using ProLong Diamond reagent (Life Technologies, Grand Island, NY), allowed to cure for 24 hours, then imaged using the EVOSfl Cell Imaging System (Thermo Fisher Scientific Inc., Waltham, MA).

For FM4-64 and DAPI co-staining, samples were prepared as described above with the following modifications. Strains GV345 (*lcpA* ^-^ p*lcpA*), GV346 (*lcpB* ^-^ p*lcpB*), GV347 [*lcpA* ^-^ (Vector)], GV348 [*lcpB* ^-^ (Vector)] and GV349 [WT (Vector)] were sub-cultured overnight in BHI broth supplemented with thiamphenicol (15μg/mL), then immobilized on poly-L-lysine coated coverslips. Samples were processed for PSII staining as described above. Following PSII staining and incubation with Alexafluor 488, cells were washed 3 times with 1% BSA and 2 times in PBS for 5 minutes each. Cells were then stained with 10 μg/mL FM4-64FX (Life Technologies, Grand Island, NY) for 30 minutes. Cells were washed 4 times in PBS then mounted on glass slides using ProLong Gold with DAPI (Life Technologies, Grand Island, NY). Slides were imaged using a Deltavision RT deconvolution fluorescence microscope (MCB Imaging Facility, University of Arizona). The scale bar represents 3.26μm, and all images presented are representative of at least three different fields.

### Biofilm studies

Biofilm assays were done as described by Pantaleon et al. [41]. Strains were grown overnight in BHI, then diluted 1:100 in BHIS supplemented with 1.8% glucose and grown for 72 hours in 24-well polystyrene plates (Costar, USA). The plate was washed two times with PBS then incubated at 37°C to dry. One milliliter of 0.2% crystal violet was added to each well, and the plate was incubated at 37°C for 30 minutes. The wells were washed twice with PBS, resuspended in 1mL of methanol/acetone (80%/20%), and the OD was measured at 570nm. Three biological replicates were performed in technical triplicate for each strain.

### Antimicrobial susceptibility testing

Susceptibility to the cathelicidin antimicrobial peptide (CAMP) LL-37 was determined for the isogenic parent (JIR8094) and the *lcpA* ^-^ mutant (GV343) by measuring the minimum inhibitory concentration (MIC) as described previously [40]. Susceptibility to the antibiotics vancomycin and metronidazole was determined by agar disk diffusion, and measuring the zone of inhibition (ZOI). Briefly, bacteria were sub-cultured to mid-exponential growth phase then normalized to an (OD)_600nm_ = 0.1. One-hundred microliters of the normalized suspension was plated as a lawn on Brucella agar (BD Biosciences, San Jose, CA), and an antibiotic disc [vancomycin (5μg/mL) or metronidazole (50μg/mL)] was placed in the center. The ZOI was measured after 24 hours of growth.

### Cytokine assessments

HT-29 cells (ATCC HTB-38) were propagated in McCoy’s 5A medium (Thermo Fisher Scientific Inc, Waltham, MA) supplemented with 10% fetal bovine serum (FBS) to 100% confluency. One day prior to stimulation, cells were incubated in serum-free medium (0.5% DMEM, 0.53% F12, 0.12% sodium bicarbonate, 1.78% HEPES and 0.5% mannose), then stimulated with 250μg of protein from surface layer extracts from JIR8094 and GV344 (*lcpB* ^-^) for 24 hours. Cell culture supernatants were collected and clarified via centrifugation to remove debris. Two independent methodologies were employed to evaluate host-cell cytokines. First, multiple cytokine/chemokine elicitation was assessed using a Proteome Profiler Human Cytokine Array Kit (R&D Systems, Minneapolis, MN). Second, a specific cytokine, Interleukin-8 (IL-8), was quantitated using the Human CXCL8/IL-8 Quantikine ELISA Kit (R&D Systems, Minneapolis, MN).

### Adhesion assays

Caco-2BBe cells (ATCC CRL-2102) were propagated in 6-well plates using Dulbecco’s Modification of Eagle’s Medium (DMEM with 4.5g/L glucose, L-glutamine and sodium pyruvate) (Corning, Corning, NY) supplemented with 10% FBS and 0.5% HEPES. One day before the assay, cells were incubated in serum-free medium (0.5% DMEM, 0.53% F12, 0.12% sodium bicarbonate, 1.78% HEPES and 0.5% mannose). Adhesion assays were performed as described by Merrigan et al. with minor modifications [42]. Briefly, strains JIR8094, GV343 (*lcpA* ^-^) and GV344 (*lcpB* ^-^) were grown overnight in BHI broth then sub-cultured in 50mL BHI, grown to an (OD)_600nm_ = 0.4, pelleted and resuspended in serum free media supplemented with 0.025M calcium chloride. Caco-2BBe cells were moved into the anaerobic chamber, serum free medium was removed from each well of the 6-well plate and 2mL of the bacterial suspension was added to each well at a multiplicity of infection (MOI) of 50. Bacteria were allowed to adhere for 40 minutes anaerobically at 37°C (the bacterial inoculum was plated on BHI plates at this point). Following incubation, bacteria were removed, Caco-2BBe cells were washed 3 times with 1mL PBS, scraped off each well, and plated on BHIS plates to enumerate percent adherence. Three biological replicates were performed in technical quadruplicate for each strain.

### Golden Syrian hamster infections

The Golden Syrian hamster model of infection was used to study the impact of *lcpA-* and *lcpB-*mutants in *C*. *difficile* virulence. Eight-week old male hamsters were orally administered clindamycin (30mg/kg; “Cleocin”, by prescription, University of Arizona Health Center Pharmacy) three days prior to infection. Five hundred spores were then also administered orally, and hamsters monitored for disease symptoms (wet-tail, ruffled coat, lethargy) throughout the course of the experiment. Moribund animals or those meeting the criteria for euthanasia were administered 270mg/kg commercial euthanizing solution (Euthanasia III, MedPharma Inc, Pomona, CA). Euthanized animals were dissected for visualization of gross pathology, and cecal contents harvested and either visualized via phase-contrast microscopy, or plated on selective medium for recovery and molecular typing of *C*. *difficile* (using 16s-23s rDNA intergenic fragment profiling and comparison with the organisms used for infection). All animal studies were approved by the Institutional Animal Care and Use Committee of the University of Arizona.

### Statistical analysis

Multiple statistical tests were employed to determine significance for experiments involving quantitation. For biofilm studies, growth and toxin assessments and adhesion assays, Student’s *t* tests were performed to compute differences between WT (Vector) and *lcpB* ^-^ (Vector), and errors bars calculated from standard deviation(s). For proteomics studies, technical replicates and standard sensitivity curve analyses were used [43], and fold change cutoffs were calculated assuming a false discovery rate (FDR) of 10%. Further, for each protein we identified, a hypergeometric test was performed to calculate a *p*-value for its abundance in the test condition compared to its abundance in the control dataset (only values ≤0.05 were accepted). A protein was classified as differentially abundant only when both FDR and p-value criteria were satisfied.

## Results

### *C*. *difficile* exhibits a capsule-like cell surface glycopolymer layer

**Fig 1:**
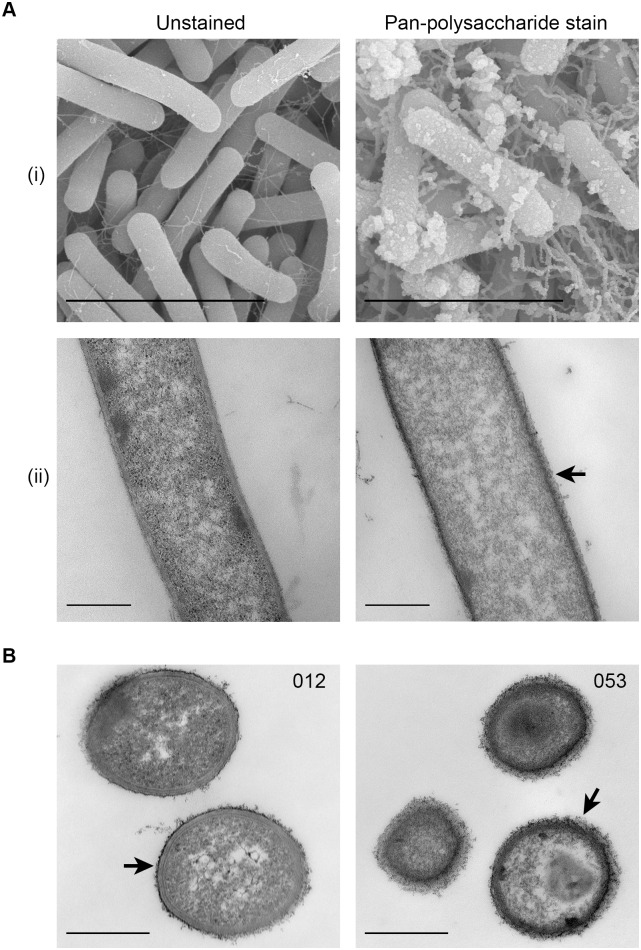
*C*. *difficile* exhibits a CWG layer. (A-i) Scanning electron micrographs of unstained (left) and ruthenium-red stained (right) *C*. *difficile* ribotype 053 strain. (A-ii) Identical to (A-i) but visualized using transmission electron microscopy. Scale bars represent 5μm for (A-i) and 500nm for (A-ii). (B) Transmission electron micrographs of ruthenium red-stained bacteria of ribotype 012 and 053 at 72 hours of growth. The scale bar represents 500nm. The black arrows point to the ruthenium red-stained CWG layer.

To investigate whether the capsular polysaccharide layer varied in clinically-relevant isolates, CWG layers were visualized in phylogenetically-diverse strains of *C*. *difficile*, including those belonging to outbreak-associated molecular types. Scanning electron microscopy (SEM) and transmission electron microscopy (TEM) of ruthenium red-strained *C*. *difficile* strain GV60 (ribotype 053; outbreak-associated) revealed the presence of an electron-dense surface CWG layer (Fig 1A). GV60 staining was markedly different from that exhibited by the *C*. *difficile* strain 630 (ribotype 012; Fig 1B), suggesting strain-to-strain variations in CWG production.

### PSII is a conserved *C*. *difficile*-specific antigen

**Fig 2:**
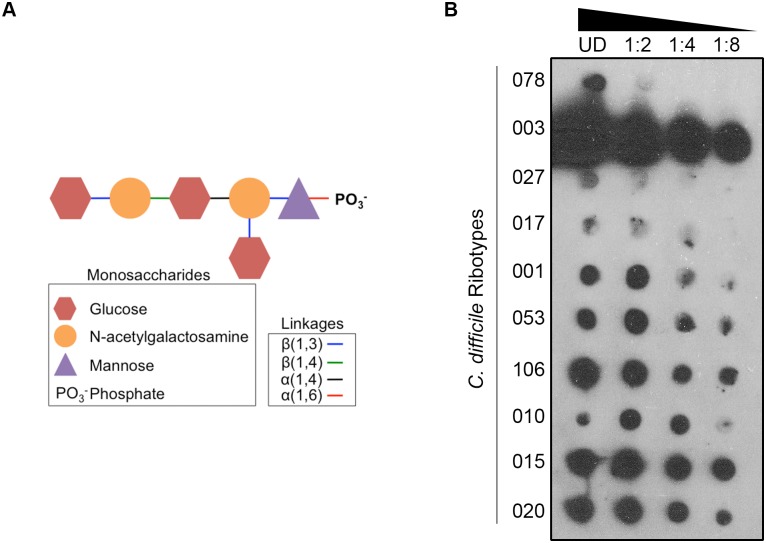
PSII is a conserved antigen across various *C*. *difficile* strains. (A) PSII structure as characterized by Ganeshapillai et al [15]. PSII is a branched, hexasaccharide repeating unit containing phosphate. (B) PSII is detected in diverse *C*. *difficile* ribotypes (UD, “undiluted”; the ramp indicates increasing to decreasing concentration from left to right).

Multiple surface-associated polysaccharides have been identified in *C*. *difficile*, and the structures of a subset of the molecules solved, including PSII (Fig 2A) [15,16]. Using a semi-quantitative PSII-specific immunoblot assay (Fig 2B), we detected varying levels of PSII in surface extracts from strains belonging to ten different *C*. *difficile* molecular types (“ribotypes”; 078, 003, 027, 017, 001, 053, 106, 010, 015, and 020; Fig 2B), but not from *Clostridium perfringens* (GV339) or *Clostridium botulinum* (S1B Fig). Purified PSII was used as a positive control, and pre-immune serum as a negative control to confirm anti-PSII antibody specificity (S1A and S1B Fig). Further, surface-localized PSII was confirmed in multiple isolates of a single *C*. *difficile* ribotype (RT027; S1B Fig). Highest PSII levels were noted for a Ribotype 003 isolate; however, this strain also demonstrated some cross-reactivity in a pre-immune serum-alone control immunoblot (S1A Fig). Thus, PSII is a common *C*. *difficile-*specific polysaccharide whose abundance varies between different clinical isolates.

### *C*. *difficile* encodes a putative CWG locus

**Fig 3:**
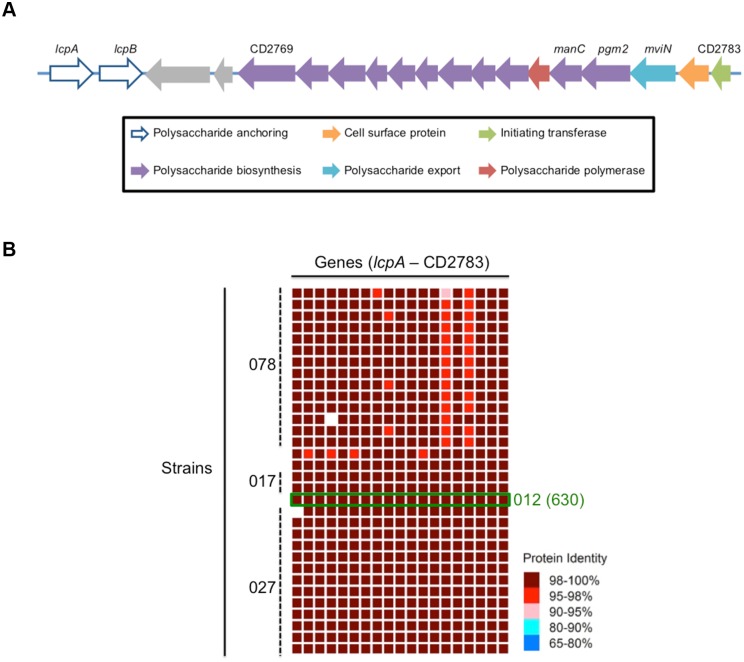
*C*. *difficile* encodes a putative CWG locus. (A) The orientation of the genes in the putative CWG locus is shown along with the generalized predicted functions of each gene product [21]. The color of each gene corresponds to its predicted function shown in the legend below the locus. (B) The locus is highly conserved at the amino acid level (95%-100%) across diverse *C*. *difficile* strains. A total of 32 clinical isolates were analyzed and compared to the reference strain CD630 (boxed in green). A list of all the strains used is provided in S1 Table.

To identify candidate genes involved in CWG biosynthesis in general, and PSII production in particular, we performed an *in silico* scan of the *C*. *difficile* strain 630 genome for ORFs with predicted glycotransferase, glycopolymer export, and glycopolymer modulatory functions. Similar to Willing et al [21], we identified an approximately 80 kilobase locus; we further observed that ORFs within this region exhibited striking similarity to the CWG biosynthetic genes of *S*. *pneumoniae* [21], including those encoding a predicted initiating transferase (CD2783), glycosyltransferases, a flippase-type exporter (MviN), a polymerase (CD2777), and proteins that anchor CWGs to the cell surface (LcpA and LcpB; Fig 3A). RT-PCR analysis of the intergenic regions between *mviN* and *pgm2*, *pgm2* and *manC*, *manC* and CD2778, and *lcpA* and *lcpB* suggests that *mviN–*CD2778 and *lcpA–lcpB* are co-transcribed (S2A Fig). The sequence-based RAST Prokaryotic Genome Annotation Server [33] revealed that this locus is highly conserved amongst phylogenetically diverse *C*. *difficile* strains (Fig 3B and S1 Table). In addition, and notably, this CWG cluster is absent in *C*. *perfringens* and *C*. *botulinum*, consistent with a putative role in the production of the *C*. *difficile*-specific antigen, PSII (Fig 2B).

### MviN depletion is associated with decreased PSII deposition

**Fig 4:**
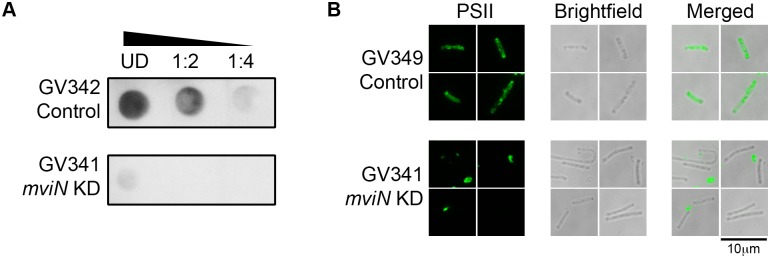
*C*. *difficile mviN* impacts cell-surface moieties. Decreased *mviN* expression in GV341 (strain expressing *mviN* anti-sense RNA: KD, “knock-down”) compared to GV342 or GV349 (vector control strains) results in less extractable PSII based on immunodot-blot analysis (A) and less PSII on the cell surface visualized by immunofluorescence microscopy (B). The ramp in (A) indicates increasing to decreasing concentration from left to right, UD is “undiluted,” and the scale bar in (B) represents 10μm.

In general, perturbation of the later steps of CWG biosynthesis, including the polysaccharide exporter, result in intracellular accumulation of toxic intermediates, or sequestration of the common lipid carrier from other essential pathways [11,44,45]. As such, attempts to disrupt the corresponding genes in other organisms have been unsuccessful [45–48]. Consistent with these observations, we were unable to disrupt five genes in the CWG biosynthetic locus (CD2783, *mviN*, CD2780, CD2779, and CD2769) via ClosTron mutagenesis. However, *mviN* expression was successfully diminished using an antisense RNA approach, and resulted in multiple phenotypes. First, *mviN*-specific antisense RNA-expressing bacteria exhibited a growth defect (S3A Fig). Second, though MviN downregulation did not affect total CWG production, there were slight morphology defects as visualized by Maneval’s capsule staining (S4 Fig). Third, specific detection and visualization of PSII revealed lower abundance of the polysaccharide in cells expressing *mviN* antisense RNA (Fig 4A). Consistent with this observation, MviN downregulation also resulted in decreased cell surface PSII staining, as visualized by immunofluorescence (Fig 4B). *mviN* is the third gene in a cluster of 19 genes, and we confirmed that is co-transcribed with its immediate downstream member *pgm2*, and that the next two genes were also each co-transcribed with their immediate neighbors (*pgm2* and *manC* and *manC* and CD2778; S2A Fig). Fourth, fully quantitative proteomic analyses also revealed that in the *mviN* knockdown strain, expression of multiple CWG gene products was decreased [including *mviN* itself (1.5 fold) and its immediate downstream genes CD2780 (1.9 fold) and CD2779 (1.6 fold); Table 2]. Expression of other, less proximal genes such as those whose products are predicted to synthesize, anchor and polymerize CWGs, was also significantly perturbed. Therefore, it is likely that multiple gene products in the CWG cluster, including MviN play a role in PSII and CWG deposition.

**Table 2:** Proteome abundance in the *mviN* knockdown strain (GV341) compared to the vector-only control strain (GV342).

Gene ID (Entrez Gene)	Predicted function/ protein product	Fold change* (log_2_ ratios; Knockdown/control)	95% CI
*lcpA* (4913178)	polysaccharide anchoring (previously LytR family transcriptional regulator)	1.32	-2.05–3.60
*lcpB* (4914621)	polysaccharide anchoring (previously LytR family transcriptional regulator)	-2.03	-3.94 – -1.06
CD2767 (4914622)	cell surface protein	-1.46	-1.87 – -1.06
CD2769 (4914624)	polysaccharide biosynthesis protein	1.04	-1.43–1.54
CD2770 (4914625)	group 1 glycosyl transferase	-1.16	-2.23–1.67
*rkpK* (4914626)	UDP-glucose 6-dehydrogenase	1.13	-1.43–1.82
*tuaG* (4914627)	family 2 glycosyl transferase	-7.66	-16.75 – -2.63
CD2773 (4914628)	family 2 glycosyl transferase	1.50	-1.11–2.42
CD2774 (4914629)	family 2 glycosyl transferase	1.33	-1.58–2.83
CD2775 (4914630)	glycerophosphotransferase	-1.17	-5.20–3.80
CD2776 (4914631)	family 2 glycosyl transferase	-1.25	-2.23–1.42
CD2777 (4914632)	membrane protein (predicted polysaccharide polymerase)	1.51	-1.38–3.19
CD2778 (4914633)	glycosyl transferase family protein	1.27	-16.44–21.48
*manC* (4915152)	mannose-1-phosphate guanylyltransferase	-1.63	-3.66–1.45
*pgm2* (4915153)	phosphoglucomutase	-1.91	-2.58 – -1.29
*mviN* (4915154)	transmembrane virulence factor (predicted polysaccharide exporter)	-1.50	-20.14–11.91
CD2782 (4915155)	cell wall binding protein	-1.13	-1.39–1.10
CD2783 (4915156)	glycosyl transferase family protein (predicted initiating transferase)	-1.25	-2.27–1.57
CD2784 (4915157)	N-acetylmuramoyl-L-alanine amidase	1.66	1.26–3.19
CD2786 (4915159)	N/A	5.60	1.26–24.89
*cwp84* (4915160)	cell surface protein	-1.21	-1.72–1.24

*All positive values signify increased amounts, and all negative values signify decreased amounts. The fold change is calculated as described in the Methods, and respective Confidence Intervals are provided for statistical inference on the variability of the data set.

### The *lcpB* mutant displays altered PSII shedding and deposition

Two genes in the CWG locus, *lcpA* and *lcpB*, were amenable to insertional inactivation, (*lcpA* ^-^ and *lcpB* ^-^, respectively) [26]. In Gram-positive organisms, LCP proteins play several roles in cell wall biosynthesis, including the anchoring of teichoic acid and/or capsular polysaccharide to the cell wall [49–54]. The function and contribution of LCP proteins to CWG biosynthesis in *C*. *difficile*, however, is unknown.

**Fig 5:**
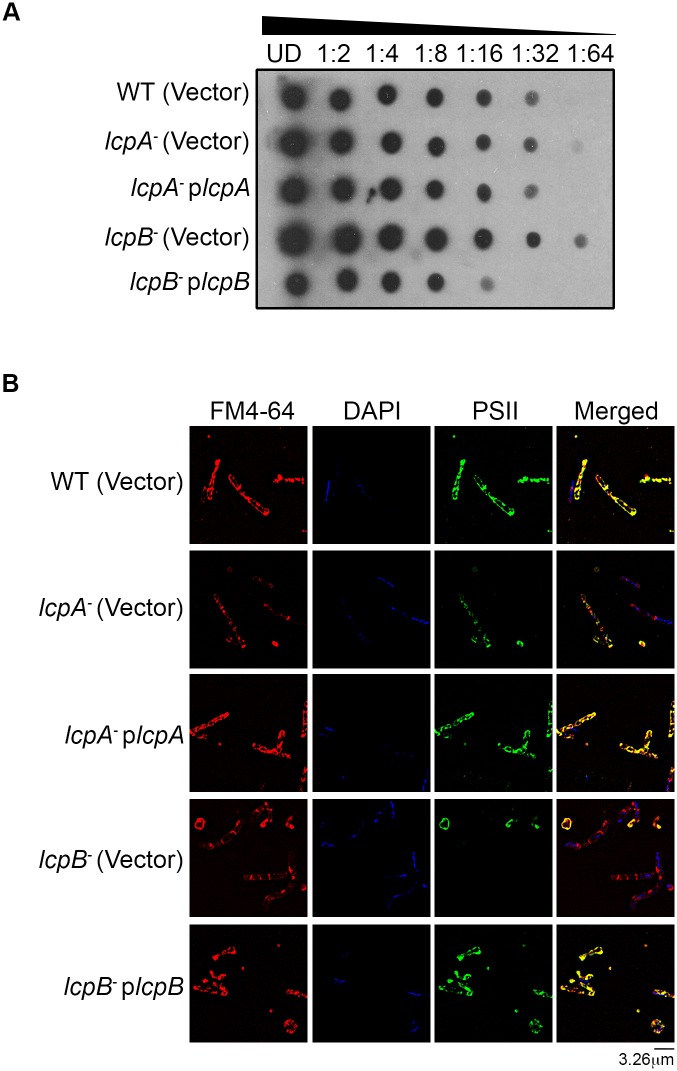
*C*. *difficile lcpB* impacts PSII production and localization. (A) Immunoblot analysis demonstrating increased shedding of PSII from the *lcpB* ^-^ strain [*lcpB* ^-^ (Vector); Row 4] compared to the isogenic parent [WT (Vector); Row 1]. Shed PSII levels are restored in a plasmid-complemented strain (*lcpB* ^-^ p*lcpB*, Row 5). Minimal increase in shed PSII from the *lcpA* ^-^ mutant [*lcpA* ^-^ (Vector); Row 2] and corresponding plasmid complementation (*lcpA* ^-^ p*lcpA*; Row 3) is also shown. UD, “undiluted”. All immunoblots are representative of a minimum of two biological replicates. (B) Immunofluorescence demonstrating PSII co-staining with the cell-surface-specific dye FM4-64 on the isogenic parent (Row 1 images). Alteration and re-localization of PSII in the *lcpA* ^-^ strain (Row 2 images) and *lcpB* ^-^ strain (Row 4 images). Altered morphology, with a curved and elongated phenotype and multiple septae are also visible in the *lcpB* ^-^ strain. Complementation-mediated restoration of PSII co-localization with FM4-64 in both mutants is shown in Rows 3 and 5 images respectively, as well as the morphology defect rescue in *lcpB* ^-^ strain (Row 5 images).

In *Staphylococcus aureus*, LCP mutants shed more capsular polysaccharide from their cell surface as a result of improper anchoring of the polymers to the cell wall [50,51]. Thus, we investigated PSII shedding to explore a possible CWG anchoring function of LcpA and LcpB in *C*. *difficile*. Relative to the parent strain, more PSII was shed from the *lcpB* ^-^ mutant; this was significantly reversed in plasmid-complemented strains (Fig 5A). Only modest changes were observed for the *lcpA* ^-^ mutant. Overall, the variation in shed PSII levels described above was not due to PSII production differences, since cytosolic PSII abundance was unchanged in parent and all mutant strains (S7 Fig).

Notably, in the parent strain, co-localization with FM464, a plasma membrane-specific dye, confirmed functional surface presentation of PSII (Fig 5B). In contrast, PSII anchoring to the cell surface was defective in both *lcpA* ^-^ and *lcpB* ^-^ mutants as evidenced by loss of co-localization with FM4-64; this was most severe in the *lcpB* ^-^ strain. Additionally, the *lcpB* ^-^ mutant also displayed altered cell morphology with curved, elongated cells (S5 Fig). Plasmid complementation of both *lcpA* ^-^ and *lcpB* ^-^ mutants restored PSII co-localization with FM4-64 similar to the parent strain, and also rescued the *lcpB* ^-^ strain morphology defect.

### The *lcpB* ^-^ mutant exhibits morphological defects

**Fig 6:**
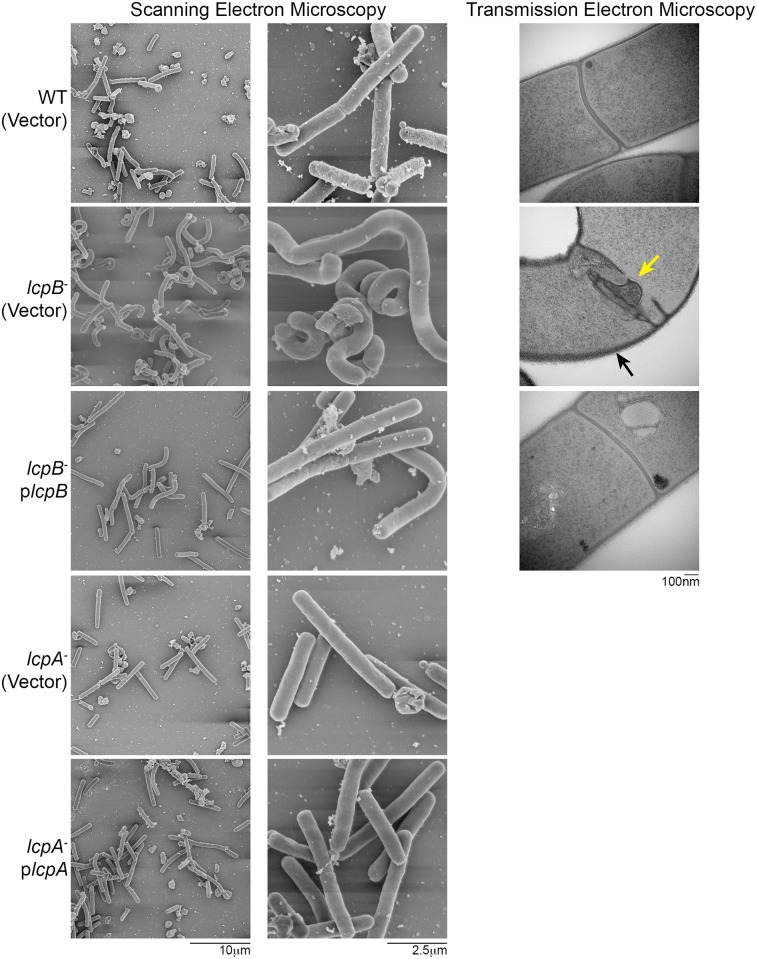
*C*. *difficile lcpB* disruption profoundly impacts bacterial morphology. Columns 1 and 2: Scanning electron micrographs of *C*. *difficile* strains in low resolution (1000X; Column 1, scale bar is 10μm), and high resolution (10,000X, Column 2, scale bar is 2.5μm). Curved, elongated morphotype of the *lcpB* ^-^ mutant [*lcpB* ^-^ (vector)] is shown in Row 2, and complementation-based rescue in Row 3. There was no obvious phenotype for the *lcpA* ^-^ strain. Column 3: Transmission electron micrographs (TEM) of parent strain (Row 1), *lcpB* ^-^ mutant [Row 2; *lcpB* ^-^ (vector)]) and complement (Row 3). Improper septum formation (yellow arrow) and a diffuse cell wall structure (black arrow) is shown. Scale bar for TEM is 100nm. All images are representative of a minimum of 10 fields visualized, and at least two biological replicate preparations.

Many bacteria have common lipid precursors for CWG and peptidoglycan biosynthesis, and cell shape is determined by appropriate deposition of each component in the cell wall [11,55]. The *lcpB* ^-^ mutant, but not the *lcpA* ^-^ mutant, displayed a modest growth defect (S3B Fig) with bacterial titers approximately one order of magnitude less than those of the parent strain at both exponential and stationary growth phases (S2 Table). Curiously, the *lcpB* ^-^ mutant also had remarkably altered morphology (S5 Fig), with cells appearing longer and thicker, harboring multiple septa, and with marked curvature; this was vividly apparent via scanning electron microscopy (Fig 6). Transmission electron microscopy of the *lcpB* ^-^ strain also revealed improper cell septum localization and structure, as well as a more diffuse cell wall. These morphological defects were significantly rescued via complementation using plasmid-encoded *lcpB*.

**Fig 7:**
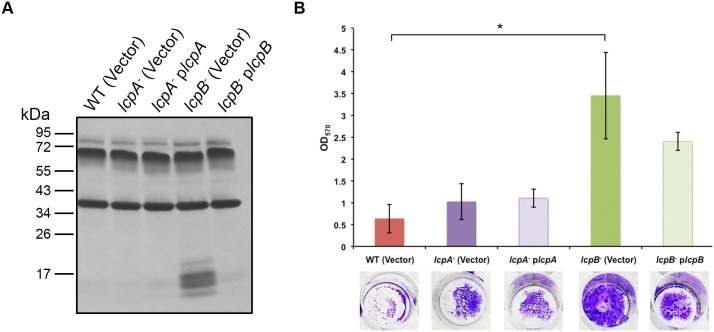
*lcpB* disruption remodels the bacterial cell surface. (A) Surface-layer Protein (SLP) profiling of *C*. *difficile* strains. Comparable SLP profiles of the isogenic parent [WT (Vector)], *lcpA* ^-^ mutant [*lcpA* ^-^ (vector)] and complement (*lcpA* ^-^ p*lcpA*; Lanes 1, 2 and 3). Altered SLP profile of the *lcpB* ^-^ mutant [*lcpB* ^-^ (vector); Lane 4] revealing additional low molecular weight products. Complementation-based restoration of the *lcpB* ^-^ mutant SLP profile (*lcpB* ^-^ p*lcpB*; Lane 5). Three independent biological replicates were performed for each strain; a representative image is shown. (B) Biofilm formation by *C*. *difficile* strains. The *lcpA* ^-^ mutant and complement produce comparable biofilms to the parent strain (Bars 1, 2 and 3), but the *lcpB* ^-^ mutant [*lcpB* ^-^ (vector)] produces a more robust biofilm, that is only partially restored to wild-type levels via plasmid complementation (Bars 4 and 5). Three biological replicates (each in technical triplicate) were performed for each strain. The error bars are represented by standard deviation estimates as well as Student’s *t* tests to compute differences between WT (Vector) and *lcpB* ^-^ (Vector). Significance is *p* < 0.05.

Cell wall remodeling and alterations were also explored in both *Lcp* mutants. First, total Surface-Layer Proteins (SLPs), integral to the *C*. *difficile* para-crystalline surface layer, were visualized. The SLP profile of *lcpA* ^-^ mutant and its complement were comparable to that of the parent strain. However, multiple low molecular weight proteins were recovered from the *lcpB* ^-^ mutant SLP extracts; these were mutant-specific since complementation with plasmid-encoded LcpB rescued the phenotype (Fig 7A). Second, biofilm formation was assessed for all strains; the *lcpB* ^-^ mutant, but not *lcpA* ^-^ mutant, produced robust biofilms compared to the parent strain, a phenotype that was partially complemented (Fig 7B). SDS-PAGE analyses are representative of a minimum of three biological replicates.

Of note, neither mutant produced altered levels of the large clostridial toxins TcdA and/or TcdB (S8 Fig). Finally, the *lcpA* ^-^ strain also exhibited no alterations in susceptibility to the antimicrobials vancomycin and metronidazole, or the cationic peptide LL-37 [measured as zone of inhibition (ZOI) and minimum inhibitory concentration (MIC) S3 Table]. The *lcpB* ^-^ strain could not be tested for these phenotypes due to a severe growth defect in the recommended MIC medium [40,56].

### The *lcp* mutants are hypervirulent in the hamster model of acute CDI

**Fig 8:**
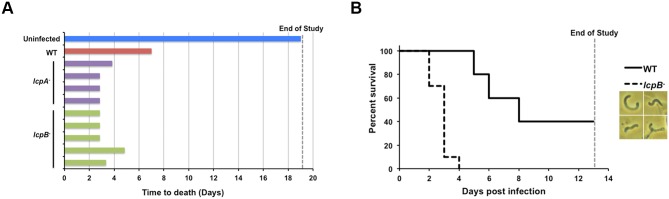
*lcp* disruption impacts *C*. *difficile* virulence. (A) Pilot study. The blue bar represents one uninfected hamster, the red bar is one wild-type infected hamster, the purple bars are *lcpA* ^-^ infected hamsters (4 total) and the green bars are *lcpB* ^-^ infected hamsters (5 total). (B) Confirmation of *lcpB* ^-^ strain hypervirulence in a powered study. A Kaplan-Meier survival plot is shown; n = 5 for parent strain-infected animals, and n = 10 for *lcpB* ^-^ mutant infected animals. Bacteria with helical morphology are visualized in the cecal contents of *lcpB* ^-^ mutant infected hamsters (inset).

Given the pleiotropic alterations in the *lcpB* ^-^ mutant, including changes to surface glycopolymer profiles, and considering that there were no differences in toxin production in the mutant compared to the parent strain, we sought to determine if cell-surface-associated phenotypes influenced *C*. *difficile* virulence in the acute Golden Syrian hamster model of infection. In a pilot study, hamsters infected with the mutants (four hamsters infected with the *lcpA* ^-^ mutant and five hamsters infected with the *lcpB* ^-^ mutant) succumbed to the infection much earlier than the isogenic parent strain-infected control (Fig 8A). This hypervirulent phenotype was confirmed for the *lcpB* ^-^ mutant in a powered study, where the average time to death for the mutant-infected hamsters (n = 10) was approximately 50% less than animals infected with the isogenic parent strain (n = 5) (Fig 8B; average time to death was 65 hours for mutant-infected hamsters, and 152 hours for wild-type infected hamsters). Additionally, cecal contents from the *lcpB* ^-^ mutant-infected hamsters yielded bacteria with helical morphology (Fig 8B, inset).

## Discussion

In this study, we confirmed the presence of a surface CWG layer in *C*. *difficile* strains belonging to diverse phylogenic groups (ribotypes). Further, our data suggest that several genes within a CWG biosynthetic locus are essential for *C*. *difficile* viability. Specifically, our studies are consistent with a key role for the putative flippase ortholog MviN, and the LytR-CpsA-Psr (LCP) ortholog LcpB, in surface deposition of the *C*. *difficile-*specific polysaccharide PSII, and in the regulation of bacterial cell shape and virulence.

*C*. *difficile* MviN displays 30% identity to its *Escherichia coli* ortholog. MviN is predicted to be a flippase, with a role in exporting lipid II for peptidoglycan biosynthesis, and is essential for viability in *E*. *coli* [57]. Consistent with a role as a flippase, *mviN* downregulation in *C*. *difficile* resulted in reduced surface PSII display. Since *mviN* appears to be co-transcribed with other member(s) of the CWG locus, and since down-regulation of *mviN* expression results in concomitantly altered abundance of other CWG locus products including LcpB, its precise contribution to the phenotypes described above remains to be established.

**Fig 9:**
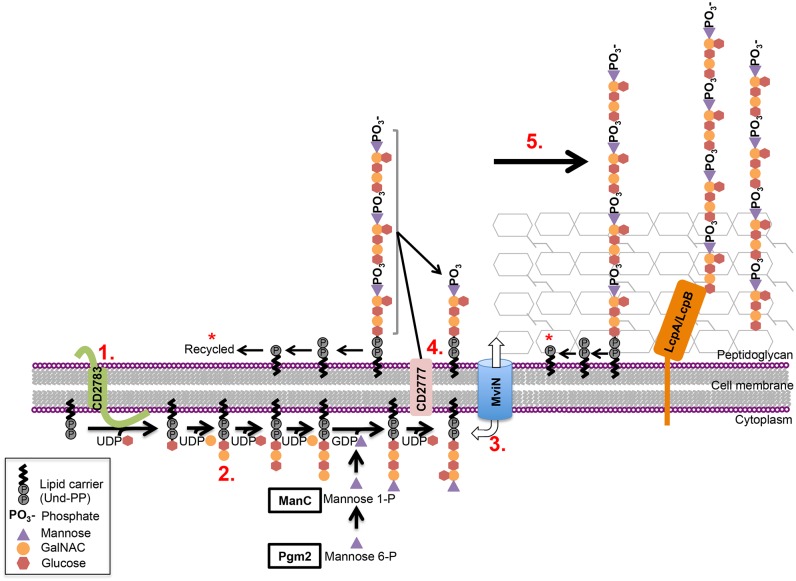
*C*. *difficile* PSII biosynthesis model [22,23,48]. One repeating unit of PSII is assembled on a lipid carrier (undecaprenyl phosphate) in the bacterial cytoplasm, exported to the cell surface, polymerized and anchored to peptidoglycan, the cell membrane, or cell wall proteins. Sequentially: (1) a predicted initiating transferase (CD2783) transfers the first sugar of the PSII repeating unit to the lipid carrier; (2) a second glycosyltransferase catalyzes the committed step of the pathway by transferring the second sugar to the repeating unit, and cytoplasmic glycosyltransferases, including ManC and Pgm2, synthesize the rest of the PSII unit; (3) a polysaccharide flippase (MviN) transports the PSII repeating unit from the cytoplasm to the cell surface; (4) a polymerase (CD2777) polymerizes the PSII chain extracellularly and the lipid carrier is recycled by an unknown mechanism (indicated by the asterisk); (5) finally, surface-anchoring factors (LcpA and/or LcpB) catalyze the transfer and anchoring of fully polymerized PSII to peptidoglycan or the cell membrane. Consistent with the NMR determinations derived in Ganeshapillai et al. [15], we have included the phosphate unit only on the mannose residue on polymerized PSII. However, the mechanistic basis of this phospho-sugar linkage has not been explored.

Of the three known CWG biosynthetic pathways, the *C*. *difficile* CWG cluster is similar to that of the streptococcal Wzx/Wzy pathway [22,45,48], which assembles complex polysaccharides, each consisting of multiple monosaccharides in a single repeating unit with a branched structure [48]. Correspondingly, PSII is comprised of repeating hexasaccharide units, and also has a branched structure [15]. The main components of the Wzx/Wzy pathway include an initiating transferase, glycosyltransferases specific for the monosaccharide units in the CWG repeating unit, a flippase-type exporter, a polymerase, and enzymes usually from the LCP protein family, that anchor the CWG to the cell surface [22,23,48]. Similar gene products, notably the putative MviN flippase, and LcpA and LcpB are encoded within the *C*. *difficile* CWG biosynthetic cluster. Further, and reminiscent of the *S*. *pneumoniae* Wzx/Wzy system, several genes in the *C*. *difficile* cluster could not be disrupted. As in *S*. *pneumoniae*, it is likely that inactivation of genes involved in the later steps of *C*. *difficile* CWG biosynthesis leads to accumulation of toxic intermediates, or sequesters common, limiting, lipid precursors in dead-end products [11,44,45]. Consistent with the apparent specificity of PSII to *C*. *difficile*, genes in the CWG locus display synteny and are highly conserved within this species, but absent in other related clostridia. Since both the *mviN-*knockdown strain and the *lcpB* ^-^ mutant display alterations in PSII deposition, the genes in this cluster likely direct multiple aspects of *C*. *difficile* CWG biology, including export. Collectively, our data, as well as the published literature, particularly in the *S*. *pneumoniae* system, support the accompanying model for PSII biosynthesis in *C*. *difficile* (Fig 9; [22]).

Recently, there has been an increasing appreciation for a direct role for LCP proteins in anchoring CWGs to the bacterial cell wall [49]. LCP proteins are widespread amongst Gram-positive bacteria, and most organisms harbor more than one homolog within their genome [49–52]. The solved crystal structure of the *S*. *pneumoniae* LCP homolog Cps2A contained tightly-bound pyrophosphoryl-lipid precursors of CWGs, suggesting a role for LCP proteins in anchoring teichoic acids (TA) and/or capsular polysaccharide (CPS) to peptidoglycan, most likely at the C6-OH linkage of N-acetylmuramic acid residues; this function has also been explored in *Staphylococcus aureus* and *Bacillus subtilis* [49–52]. All three organisms encode multiple LCP homologs in their genomes, with partial functional redundancy. While single LCP mutants displayed modest defects, double- and triple-LCP mutants shed CPS and/or TAs into the medium, and had severe growth and morphology defects [49–52]. In contrast, our data suggest non-redundant roles for *C*. *difficile* LcpA and LcpB, despite 64% amino acid identity. Relative to the *lcpB* ^-^ strain, the *lcpA* ^-^ strain displayed modest alterations, particularly in the in vitro phenotypes. Given the gene organization and RT-PCR analysis (S2B Fig), it is likely that the *lcpA* ^-^ defects are due to polar effects on LcpB expression. In contrast, the *lcpB* ^-^ strain displayed a range of phenotypes, which were at least partly, if not fully, complemented by plasmid-encoded *lcpB*.

The *lcpB* ^-^ mutant sheds more PSII into the supernatant compared to its isogenic parent, and this results in little to no PSII surface staining; therefore, we conclude that LcpB is required for maintenance of deposited PSII on the bacterial surface. It is as yet unclear where PSII is anchored; a recent study reveals roles for LCP proteins beyond peptidoglycan-mediated anchoring [53].

In addition to a defect in PSII biosynthesis and deposition, the *lcpB* ^-^ mutant also displays significant morphological alteration(s). In *Bacillus anthracis*, one of six LCP homologs (*lcpD*) is required for proper attachment of CWGs to peptidoglycan. However, LcpD, in concert with another homolog LcpB3, is also involved in maintaining cell size and proper chain length formation [54]. The authors speculated that this extra role could be due to differences in cell morphology between cocci versus bacilli, as bacilli require extra machinery to maintain their rod shape, whereas cocci do not. Interestingly, *mviN-*knockdown also impacted cell morphology, resulting in curved cells (S4 Fig). It is possible that CWG biosynthetic genes, including LCP proteins are associated with enzymes involved in maintaining proper rod-shape, and that disruption of the corresponding genes may lead not only to improper anchoring of CWGs, but also a defect in peptidoglycan deposition and/or rod shape formation [49]. We therefore propose that *lcpB* disruption perturbs PSII deposition as well as other processes in cell wall biosynthesis.

Given the pleotropic CWG- and morphology-altering impacts of *lcpB* disruption, the marked hypervirulence of the *lcpB* ^-^ mutant (as well as *lcpA* ^-^ strain) in hamsters was unexpected. The increased virulence could not be attributed to alterations in toxin levels since the amount of toxin produced by the mutants was not significantly different from the isogenic parent strain. Further, the presence of the *ermB* cassette cannot be considered to be a contributing factor toward virulence since published mutants in the JIR8094 background have displayed both hypervirulent [58] and avirulent [59–61] phenotypes in the same hamster model of infection. There are several other possible explanations for the heightened virulence of our mutants, all implicating a role for non-toxin virulence factors. First, reduced surface capsular polysaccharides (CPS) may influence bacterial adherence to epithelial cells [62,63]. *Streptococcus pneumoniae* colonizes more efficiently when it produces less CPS, possibly due to enhanced attachment to epithelial cells via exposed adhesins [32]. We performed adhesion assays to Caco-2BBe human intestinal epithelial cells [42] but observed either no difference (*lcpA* ^-^ mutant) in adhesion compared to the parent strain, or decreased adhesion (*lcpB* ^-^ mutant; S9A Fig). Thus, it is unlikely that epithelial cell adhesion contributes to the increase in virulence in either mutant. Second, the altered surface morphology may facilitate biofilm formation [64]. Indeed, our studies revealed robust biofilm formation by the *lcpB* ^-^ strain (Fig 7B), but it is unlikely that this phenotype profoundly influences virulence since hamster lethality was observed in less than 48 hours. Third, and most plausible for this study, depletion of CWGs may uncover pro-inflammatory bacterial ligands, as has been noted for the unencapsulated *S*. *suis* mutants [64]. Thus, the lack of cell surface PSII deposition in the *lcpB* ^-^ strain could expose other, pro-inflammatory, surface factors. The *lcpB* ^-^ mutant displays an altered cell wall protein profile (Fig 7A), specifically the presence of lower molecular weight products that are absent in the parent strain extracts. Consistent with the display of altered ligands in the mutants, human intestinal epithelial cells treated with surface layer extracts from the *lcpB* ^-^ strain secreted increased amounts of the pro-inflammatory cytokine Interleukin-8 (IL-8; S9B Fig; two independent methodologies). Fourth, a remodeled cell surface may alter susceptibility to killing by host antimicrobial peptides, a first line of defense during infection. The *lcpA* ^-^ strain showed similar susceptibility as the parent strain to LL-37, an intestine-specific cathelicidin antimicrobial peptide ([40]; S3 Table; the *lcpB* ^-^ strain could not be tested because it failed to grow robustly on the approved testing medium). Finally, increased shedding of PSII may be directly hyper-immunostimulatory and thus detrimental. However this is relevant only to the *lcpB* ^-^ mutant, and therefore cannot be invoked as a common mechanism for increased virulence. Future studies will aim to parse between the above possibilities.

For all the results described in this work, we employed the *C*. *difficile* strain JIR8094, an erythromycin-sensitive derivative of the clinical isolate CD630. The primary rationale for this was two-fold. First, strain 630 and its derivatives have a more prolonged lethality profile in the hamster model of CDI compared with other isolates (range 4–7 days; [7,59,60]), thus enabling robust time-to-death estimations, and second, multiple molecular assessments have been performed in this strain background allowing for comparison between published studies. However, as proof-of-principle, *lcpB* was also disrupted in a ribotype 027 (outbreak-associated) isolate, as well as a non-toxigenic strain of *C*. *difficile* (S10 Fig). In both cases, preliminary bright-field microscopy visualizations revealed altered bacterial morphology similar to that observed with the JIR8094 *lcpB* ^-^ strain (Fig 6).

In conclusion, we have shown that proteins encoded by the *C*. *difficile* CWG locus, especially LcpB, influence PSII shedding, cell shape, and virulence. Future studies will focus on elucidating the steps involved in PSII attachment to the cell wall, as well as an investigation of the impact of cell morphology and cell surface composition on *C*. *difficile*-induced host immune responses. PSII has been proposed as a vaccine candidate for CDI prevention, and several studies have assessed its immunogenic and protective potential [18,19,65,66]. Since PSII, and possibly other CWGs, fundamentally affect key aspects of *C*. *difficile* pathogenesis, it is imperative to understand CWG biosynthetic mechanisms to optimally engineer next-generation anti-CDI biotherapeutics and other potential vaccine candidates.

## Supporting Information

S1 Table Strains used in computing protein identities in the CWG locus for Fig 3B. Genome sequences were either obtained from GenBank (NCBI; [34]) or from the Vedantam Laboratory strain collection.
(DOCX)

S2 Table Bacterial titers were determined during growth curves for the *lcpA* ^-^ and *lcpB* ^-^ mutants (shown in S3B Fig) at two time points: mid-log and stationary phase. The bacterial counts are represented by CFU/mL and three biological replicates were performed for each strain in each set. A Student’s *t* test was performed at each time-point for both data sets and the results are only significant between the *lcpB* ^-^ mutant and wild-type strain at both mid-log and stationary time points (*p* < 0.05).
(DOCX)

S3 Table LL-37 susceptibility for the isogenic parent (JIR8094) and *lcpA* ^-^ mutant strain. A scrambled LL-37 control is also shown. The numbers are representative of 1 biological replicate for the isogenic parent and 2 biological replicates for the *lcpA* ^-^ mutant. Susceptibility to vancomycin and metronidazole (ZOI measurements). The numbers are representative of 3 biological replicates for the isogenic parent, and 2 biological replicates for the *lcpA* ^-^ mutant.
(DOCX)

S1 Fig Anti-PSII antiserum specificity. (A) Purified PSII was used as a positive control. Following CWG extraction using the EDTA-TEA method, EDTA-TEA buffer was probed with PSII-LTB rabbit antiserum (top) and extracts from various strains were probed with pre-immune serum to verify lack of cross-reactivity (bottom). Ribotype 003 exhibited slight cross-reactivity with the pre-immune serum, but the amount is negligible compared to the total amount of PSII detected in Fig 2B. (B) PSII was also detected from other 027 ribotypes as well as *Clostridium perfringens* and *Clostridium botulinum*. PSII-LTB rabbit antiserum reacts robustly with all 027 strains tested but not *C*. *perfringens* or *C*. *botulinum* (top). These strains were also probed with pre-immune serum to verify lack of cross-reactivity (bottom). The REA types of the 027 strains tested are as follows: 027–1 = BI-1 (GV44), 027–2 = BI-6 (GV45), 027–3 = BI-8 (GV46), 027–4 = BI-17 (GV48), and 027–5 = BI-23 (GV51) (UD, “undiluted”; the ramps indicate increasing to decreasing concentration from left to right).
(TIF)

S2 Fig RT-PCR analysis of the intergenic regions in the CWG locus. RNA was extracted from JIR8094 mid-log cultures, DNAse treated, reverse-transcribed and the intergenic regions between *mviN* and *pgm2*, *pgm2* and *manC*, *manC* and CD2778, and *lcpA* and *lcpB* were amplified by conventional PCR. The DNA controls use genomic DNA from 630ΔErm and the negative controls have no template. “No RT” controls indicate no reverse transcription for those samples. Schematics of the regions that were amplified are shown in (A) and (B) and denoted by the colored arrows. There are amplicons for all pairs tested suggesting that *mviN*–CD2778 (A) and *lcpA–lcpB* (B) respectively, are co-transcribed.
(TIF)

S3 Fig Growth kinetics for GV341 and the *lcpA* ^-^ and *lcpB* ^-^ mutants. (A) The *mviN* asRNA knockdown strain (GV341) displays a growth defect. Biological duplicates of each strain and condition (with or without induction) are depicted. (B) The *lcpA* ^-^ mutant displays similar growth kinetics to the wild-type strain (top), but the *lcpB* ^-^ mutant displays a slight growth defect (bottom). Three biological replicates were performed for each strain in each set. The bacterial titers were determined at mid-log and stationary phase time points and are presented in S2 Table. The error bars denote standard deviation.
(TIF)

S4 Fig Maneval’s capsule staining of the *mviN* knockdown strain. Maneval’s capsule staining was performed for the *mviN* knockdown strain. While there is no apparent difference in total CWG on the *mviN* knockdown strain (*mviN* KD) compared to the control strain (Control), there are slight alterations in cell morphology in the knockdown strain.
(TIF)

S5 Fig Alternate visualization of *lcpB* ^-^ morphology defects. Maneval’s capsule staining was performed for the *lcpA* ^-^ and *lcpB* ^-^ mutants (A). Brightfield images were taken using the EVOSfl microscope for WT (Vector), *lcpB* ^-^ (Vector) and *lcpB* ^-^ p*lcpB* (B). There is noticeable alteration in cell morphology of the *lcpB* ^-^ but not *lcpA* ^-^ mutant compared to the wild-type strain (A), and this difference is partially complemented with a plasmid-encoded copy of *lcpB* (B).
(TIF)

S6 Fig Normalization for PSII immunoblots. Fifty micrograms of total protein from the “shed” (supernatant) fraction of WT (Vector), *lcpA* ^-^ (Vector), *lcpA* ^-^ p*lcpA*, *lcpB* ^-^ (Vector) and *lcpB* ^-^ p*lcpB* were run on a 4–20% TGX gel and stained with Gel Code Blue. The staining was evenly distributed between all strains indicating normalized protein in the “shed proteins” fraction. A representative gel is shown for a total of two biological replicates for each strain.
(TIF)

S7 Fig Cytosolic PSII estimations. Serial dilution of one microgram of total protein from the cytosolic fraction of WT (Vector)—Row 1, *lcpA* ^-^ (Vector)–Row 2, *lcpA* ^-^ p*lcpA*–Row 3, *lcpB* ^-^ (Vector)–Row 4, and *lcpB* ^-^ p*lcpB*–Row 5. PSII levels determined by immunoblotting. A representative blot is shown (reflective of 2 biological replicates for each strain (UD, “undiluted”; the ramp indicates increasing to decreasing PSII amounts from left to right).
(TIF)

S8 Fig Toxin analyses for the *lcpA* ^-^ and *lcpB* ^-^ mutants. Both the *lcpA* ^-^ (A) and *lcpB* ^-^ (B) mutants produce similar levels of toxin compared to the isogenic parent strain control. Data are normalized to total protein in the supernatant (represented by arbitrary units, AU). Three biological replicates were performed for each strain in each set. A Student’s *t* test was performed and indicated no significance between any of the strains (*p* > 0.05). The error bars denote standard deviation.
(TIF)

S9 Fig Epithelial cell adhesion assays and IL-8 analysis. (A) Adhesion to Caco-2BBe epithelial cells was determined in the *lcpA* ^-^ and *lcpB* ^-^ mutants compared to the wild-type strain. The target MOI was 50 for the wild-type and *lcpA* ^-^ mutant, but 100 was used for the *lcpB* ^-^ mutant due to variability in percent adhesion with lower MOIs. The percent adhesion of both mutants trended less than the wild-type strain, but a Student’s *t* test indicate that the differences are not significant in either mutant (*p* > 0.05). A total of three biological replicates were done four times for each strain and error bars represent standard deviation. (B) IL-8 secretion from HT-29 epithelial cells was determined by stimulation of HT-29’s with 250μg of total protein from surface layer extracts from the *lcpB* ^-^ mutant and wild-type strains. Data from two different methodologies are shown (ELISA, bar graph, 1 biological replicate; and Cytokine Profiler Array, immunoblot, 1 biological replicate); purified flagellin from *Salmonella typhimurium* is the positive control (blue bar), and all samples were normalized using no-protein control.
(TIF)

S10 Fig *lcpB* ^-^ mutant morphotypes in multiple *C*. *difficile* ribotype backgrounds. Top and bottom left panels: Parent (top; GV44; “WT”) and *lcpB* ^-^ (bottom; GV435) derivative, *C*. *difficile* ribotype 027 strain. Top and bottom right panels: Parent (top; GV66, “WT”) and *lcpB* ^-^ (bottom; GV436) derivative, non-toxigenic strain. All morphology visualized using Maneval’s capsule staining (described in Methods).
(TIF)
