# Supplementary material for: A Clostridium difficile Cell Wall Glycopolymer Locus Influences Bacterial Shape, Polysaccharide Production and Virulence
Source: PLoS Pathog. 2016 Oct 14;12(10):e1005946. doi: 10.1371/journal.ppat.1005946 (PMC5065235; doi:10.1371/journal.ppat.1005946)
Supplement: S1 Table — Genome sequences were either obtained from GenBank (NCBI; [34]) or from the Vedantam Laboratory strain collection. (DOCX) [file ppat.1005946.s001.docx]

| **Strain ID** | **Accession number** |
| --- | --- |
| 6466 | ADDE01000060 |
| QCD23m63 | CM000660 |
| JGS790 | N/A; Vedantam collection |
| NAP08 | NZ_GG770710 |
| NAP07 | NZ_GG770749 |
| JGS6176 | N/A; Vedantam collection |
| JGS6193 | N/A; Vedantam collection |
| M120 | FN665653 |
| JGS382 | N/A; Vedantam collection |
| JGS860 | N/A; Vedantam collection |
| JGS1004 | N/A; Vedantam collection |
| JGS980 | N/A; Vedantam collection |
| JGS817 | N/A; Vedantam collection |
| JGS918 | N/A; Vedantam collection |
| 6534 | ADEJ01000893 |
| 6503 | ADEI01000027 |
| CF5 | FN665652 |
| M68 | FN668375 |
| 630 | AM180355 |
| ATCC43255 | CM000604 |
| BI9 | FN668944 |
| QCD63q42 | CM000637 |
| BI1 | FN668941 |
| QCD76w55 | CM000661 |
| QCD37x79 | CM000658 |
| QCD32g58 | CM000287 |
| CIP107932 | CM000659 |
| QCD97b34 | CM000657 |
| 196 | NZ_AVIE01000310 |
| R20291 | FN545816 |
| 2007855 | FN665654 |
| QCD66c26 | CM000441 |
