# Supplementary material for: A Clostridium difficile Cell Wall Glycopolymer Locus Influences Bacterial Shape, Polysaccharide Production and Virulence
Source: PLoS Pathog. 2016 Oct 14;12(10):e1005946. doi: 10.1371/journal.ppat.1005946 (PMC5065235; doi:10.1371/journal.ppat.1005946)
Supplement: S2 Table — The bacterial counts are represented by CFU/mL and three biological replicates were performed for each strain in each set. A Student’s t test was performed at each time-point for both data sets and the results are only significant between the lcpB - mutant and wild-type strain at both mid-log and stationary time points (p < 0.05). (DOCX) [file ppat.1005946.s002.docx]

| **Strain** | **WT vs. *lcpA^-^*** | | **WT vs. *lcpB^-^*** | |
| --- | --- | --- | --- | --- |
|  | **Mid-log titer CFU/mL (3h)** | **Stationary titer CFU/mL (8h)** | **Mid-log titer CFU/mL (4h)** | **Stationary titer CFU/mL (9h)** |
| JIR8094 (WT) | 1.52 x 10^8^ | 2.83 x 10^8^ | 6.33 x 10^7^ | 2.93 x 10^8^ |
| *lcpA^-^* | 1.02 x 10^8^ | 2.43 x 10^8^ | N/A | N/A |
| *lcpB^-^* | N/A | N/A | 4.17 x 10^6^ | 9.00 x 10^7^ |
