# Supplementary material for: A Clostridium difficile Cell Wall Glycopolymer Locus Influences Bacterial Shape, Polysaccharide Production and Virulence
Source: PLoS Pathog. 2016 Oct 14;12(10):e1005946. doi: 10.1371/journal.ppat.1005946 (PMC5065235; doi:10.1371/journal.ppat.1005946)
Supplement: S3 Table — A scrambled LL-37 control is also shown. The numbers are representative of 1 biological replicate for the isogenic parent and 2 biological replicates for the lcpA - mutant. Susceptibility to vancomycin and metronidazole (ZOI measurements). The numbers are representative of 3 biological replicates for the isogenic parent, and 2 biological replicates for the lcpA - mutant. (DOCX) [file ppat.1005946.s003.docx]

| **Strain** | **MIC (μg/mL)** | | **ZOI (mm)** | |
| --- | --- | --- | --- | --- |
|  | **LL-37** | **Scrambled LL-37** | **Vancomycin** | **Metronidazole** |
| JIR8094 (WT) | 8 | >64 | 17 | 41 |
| *lcpA^-^* | 8 | >64 | 18 | 41 |
| *lcpB^-^* | N.D. | N.D. | N.D. | N.D. |
