# Supplementary figures and images for: A Clostridium difficile Cell Wall Glycopolymer Locus Influences Bacterial Shape, Polysaccharide Production and Virulence
Source: PLoS Pathog. 2016 Oct 14;12(10):e1005946. doi: 10.1371/journal.ppat.1005946 (PMC5065235; doi:10.1371/journal.ppat.1005946)

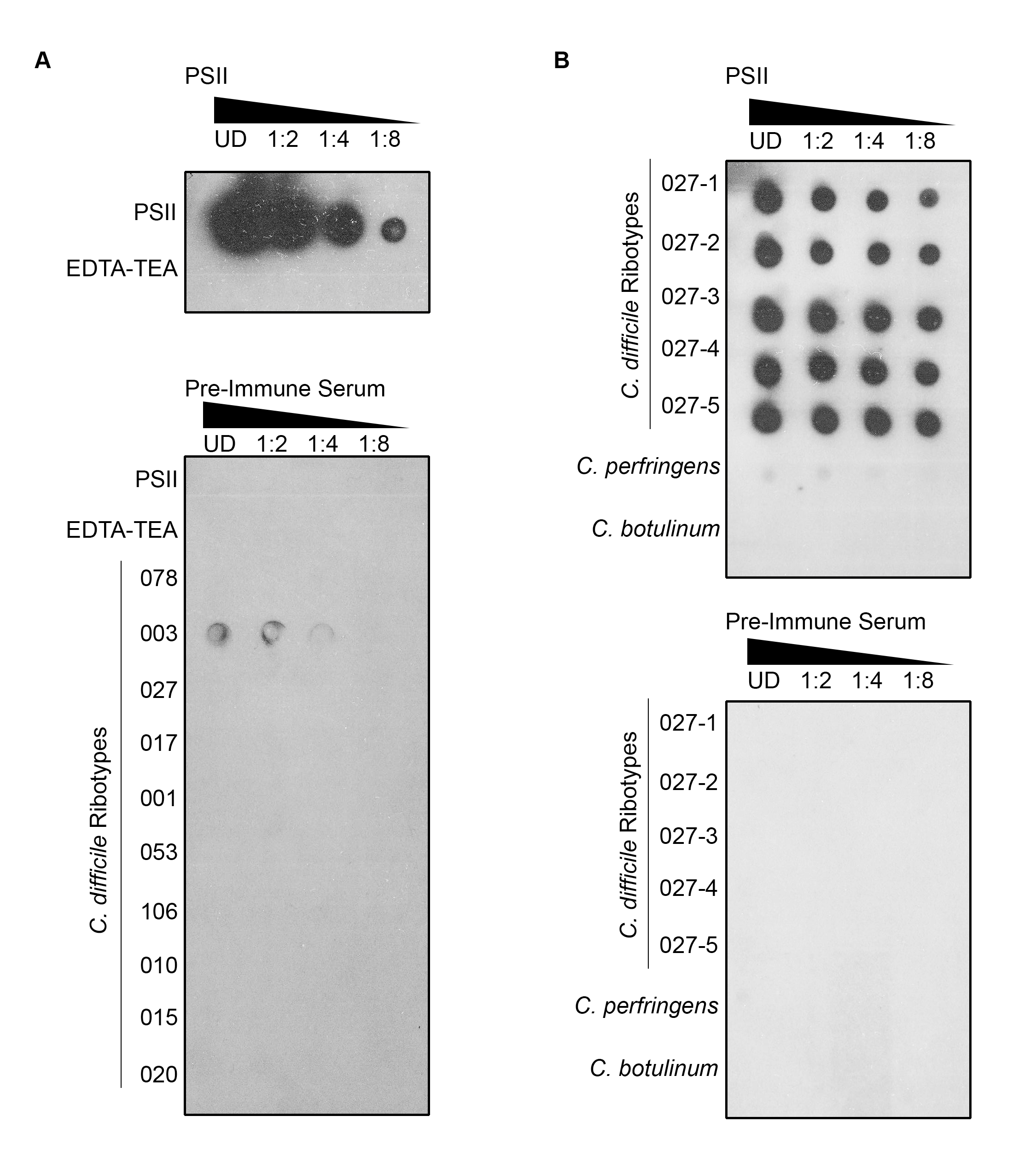

Supplement: S1 Fig — (A) Purified PSII was used as a positive control. Following CWG extraction using the EDTA-TEA method, EDTA-TEA buffer was probed with PSII-LTB rabbit antiserum (top) and extracts from various strains were probed with pre-immune serum to verify lack of cross-reactivity (bottom). Ribotype 003 exhibited slight cross-reactivity with the pre-immune serum, but the amount is negligible compared to the total amount of PSII detected in Fig 2B. (B) PSII was also detected from other 027 ribotypes as well as Clostridium perfringens and Clostridium botulinum. PSII-LTB rabbit antiserum reacts robustly with all 027 strains tested but not C. perfringens or C. botulinum (top). These strains were also probed with pre-immune serum to verify lack of cross-reactivity (bottom). The REA types of the 027 strains tested are as follows: 027–1 = BI-1 (GV44), 027–2 = BI-6 (GV45), 027–3 = BI-8 (GV46), 027–4 = BI-17 (GV48), and 027–5 = BI-23 (GV51) (UD, “undiluted”; the ramps indicate increasing to decreasing concentration from left to right). (TIF) [file ppat.1005946.s004.tif]

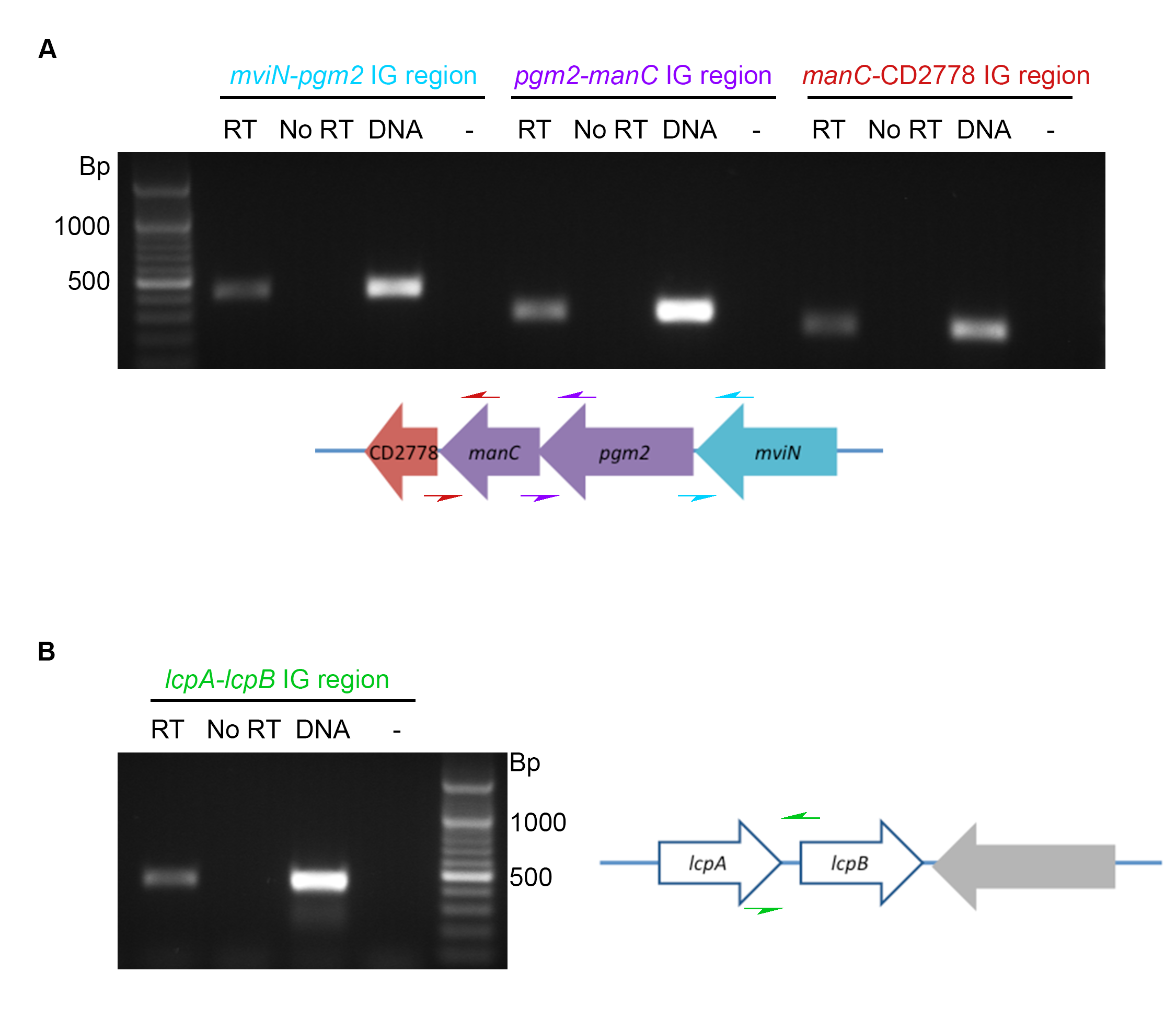

Supplement: S2 Fig — RNA was extracted from JIR8094 mid-log cultures, DNAse treated, reverse-transcribed and the intergenic regions between mviN and pgm2, pgm2 and manC, manC and CD2778, and lcpA and lcpB were amplified by conventional PCR. The DNA controls use genomic DNA from 630ΔErm and the negative controls have no template. “No RT” controls indicate no reverse transcription for those samples. Schematics of the regions that were amplified are shown in (A) and (B) and denoted by the colored arrows. There are amplicons for all pairs tested suggesting that mviN–CD2778 (A) and lcpA–lcpB (B) respectively, are co-transcribed. (TIF) [file ppat.1005946.s005.tif]

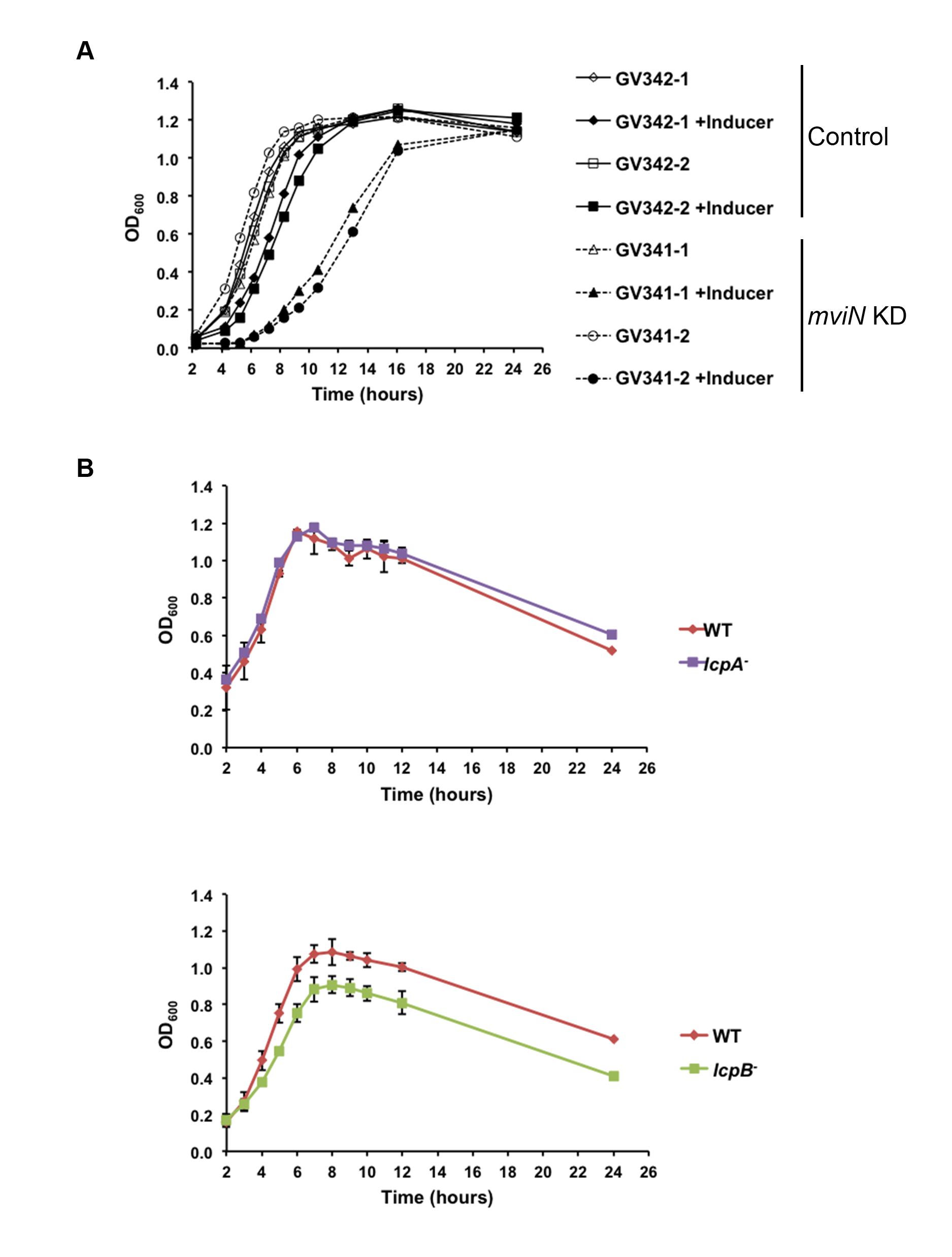

Supplement: S3 Fig — (A) The mviN asRNA knockdown strain (GV341) displays a growth defect. Biological duplicates of each strain and condition (with or without induction) are depicted. (B) The lcpA - mutant displays similar growth kinetics to the wild-type strain (top), but the lcpB - mutant displays a slight growth defect (bottom). Three biological replicates were performed for each strain in each set. The bacterial titers were determined at mid-log and stationary phase time points and are presented in S2 Table. The error bars denote standard deviation. (TIF) [file ppat.1005946.s006.tif]

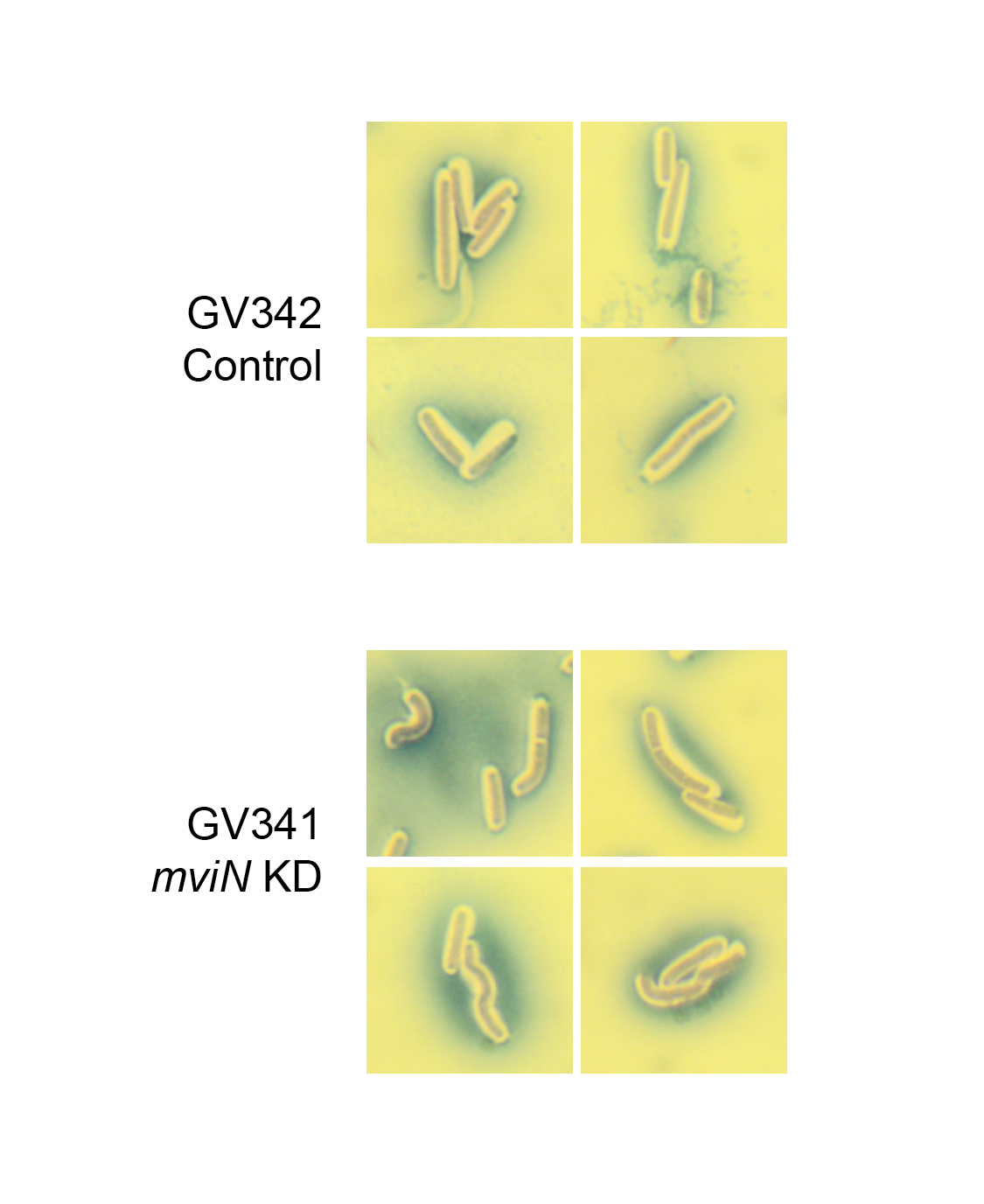

Supplement: S4 Fig — Maneval’s capsule staining was performed for the mviN knockdown strain. While there is no apparent difference in total CWG on the mviN knockdown strain (mviN KD) compared to the control strain (Control), there are slight alterations in cell morphology in the knockdown strain. (TIF) [file ppat.1005946.s007.tif]

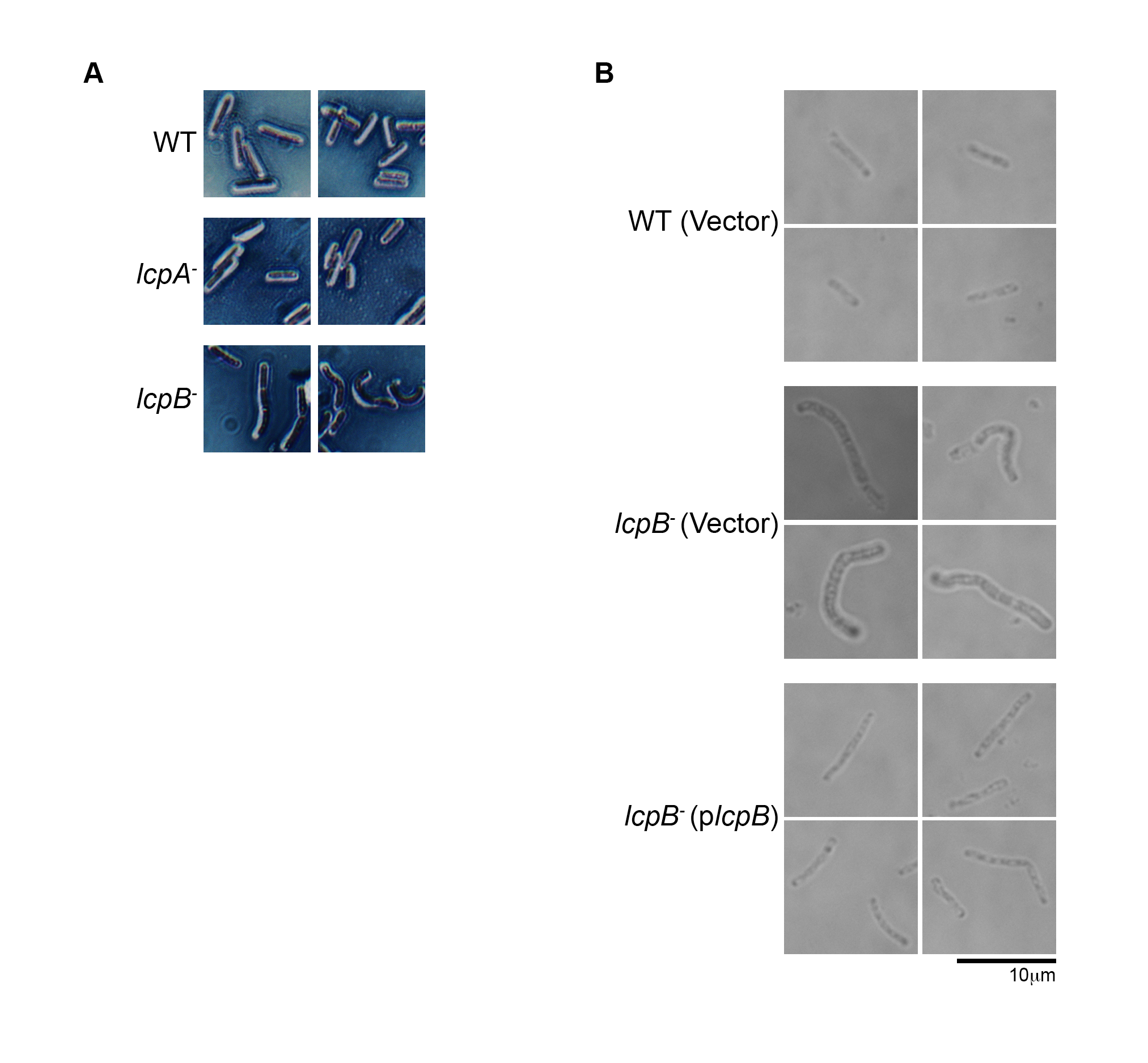

Supplement: S5 Fig — Maneval’s capsule staining was performed for the lcpA - and lcpB - mutants (A). Brightfield images were taken using the EVOSfl microscope for WT (Vector), lcpB - (Vector) and lcpB - plcpB (B). There is noticeable alteration in cell morphology of the lcpB - but not lcpA - mutant compared to the wild-type strain (A), and this difference is partially complemented with a plasmid-encoded copy of lcpB (B). (TIF) [file ppat.1005946.s008.tif]

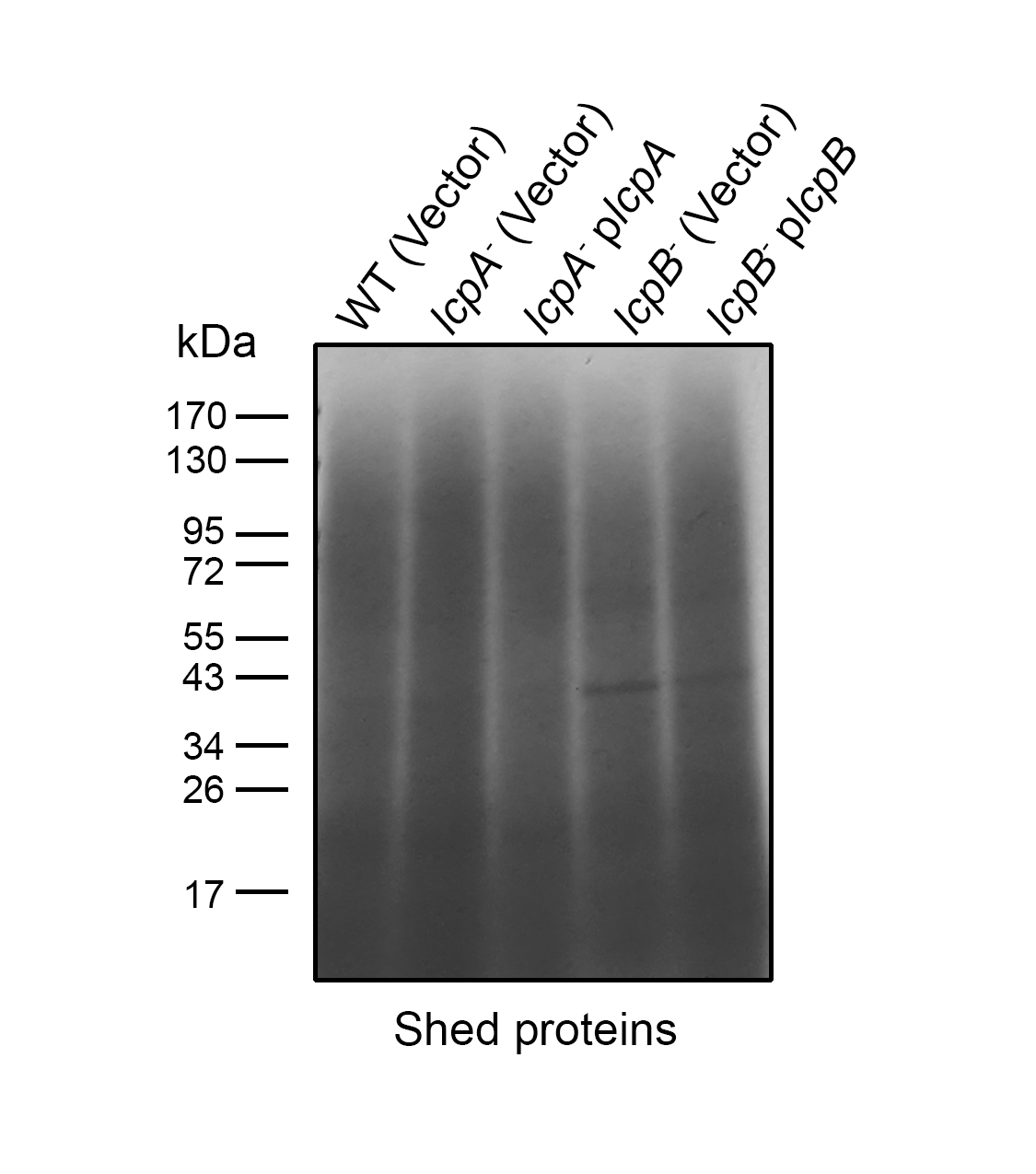

Supplement: S6 Fig — Fifty micrograms of total protein from the “shed” (supernatant) fraction of WT (Vector), lcpA - (Vector), lcpA - plcpA, lcpB - (Vector) and lcpB - plcpB were run on a 4–20% TGX gel and stained with Gel Code Blue. The staining was evenly distributed between all strains indicating normalized protein in the “shed proteins” fraction. A representative gel is shown for a total of two biological replicates for each strain. (TIF) [file ppat.1005946.s009.tif]

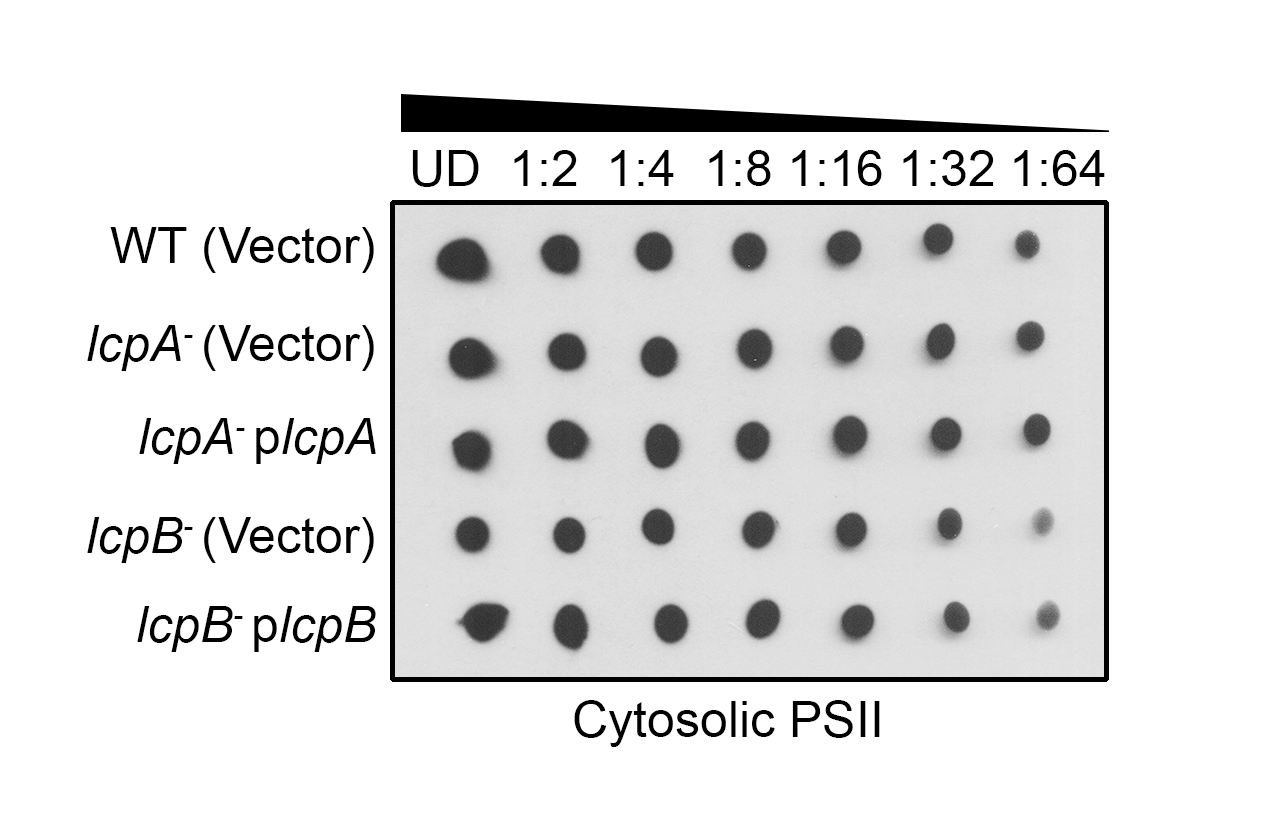

Supplement: S7 Fig — Serial dilution of one microgram of total protein from the cytosolic fraction of WT (Vector)—Row 1, lcpA - (Vector)–Row 2, lcpA - plcpA–Row 3, lcpB - (Vector)–Row 4, and lcpB - plcpB–Row 5. PSII levels determined by immunoblotting. A representative blot is shown (reflective of 2 biological replicates for each strain (UD, “undiluted”; the ramp indicates increasing to decreasing PSII amounts from left to right). (TIF) [file ppat.1005946.s010.tif]

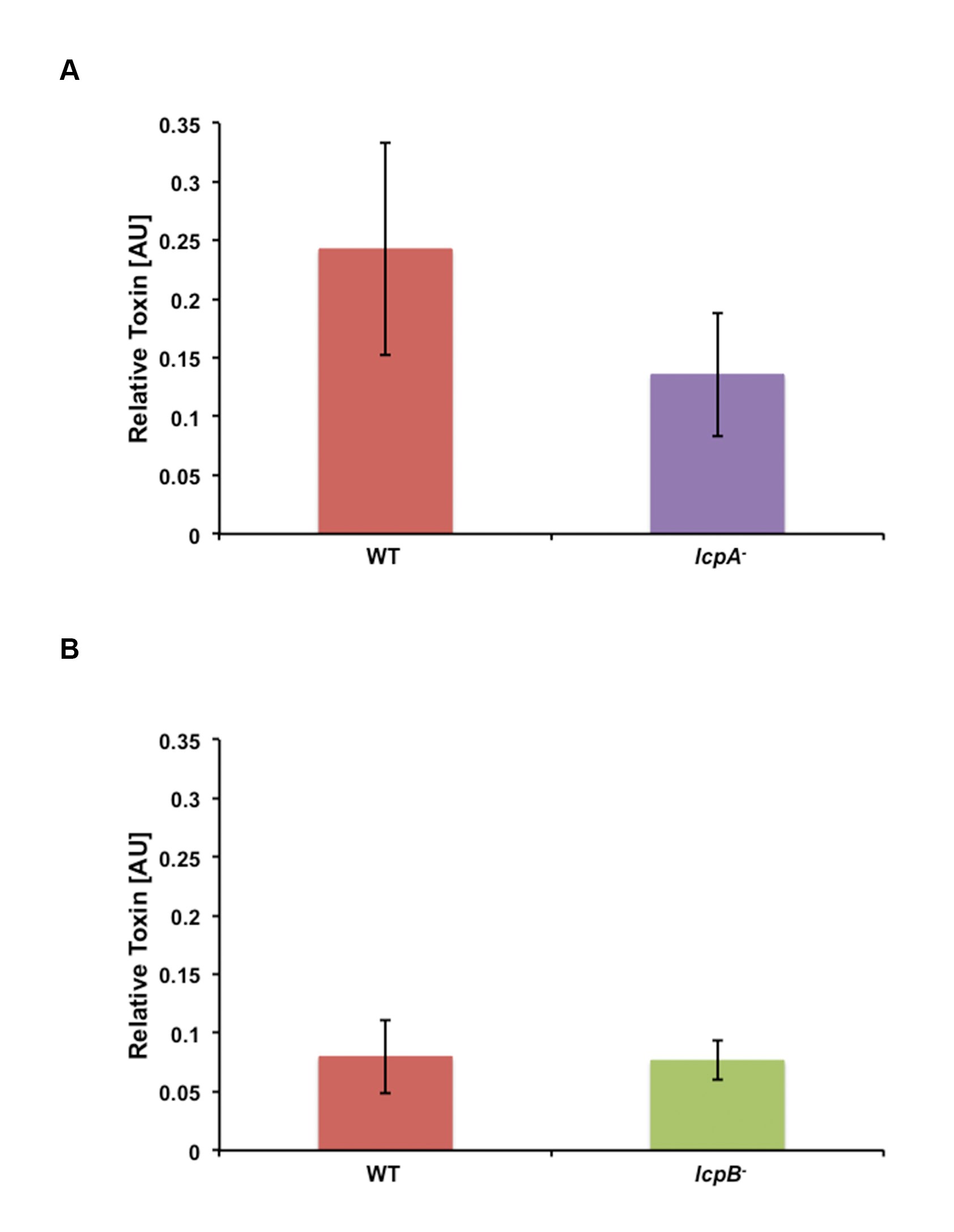

Supplement: S8 Fig — Both the lcpA - (A) and lcpB - (B) mutants produce similar levels of toxin compared to the isogenic parent strain control. Data are normalized to total protein in the supernatant (represented by arbitrary units, AU). Three biological replicates were performed for each strain in each set. A Student’s t test was performed and indicated no significance between any of the strains (p > 0.05). The error bars denote standard deviation. (TIF) [file ppat.1005946.s011.tif]

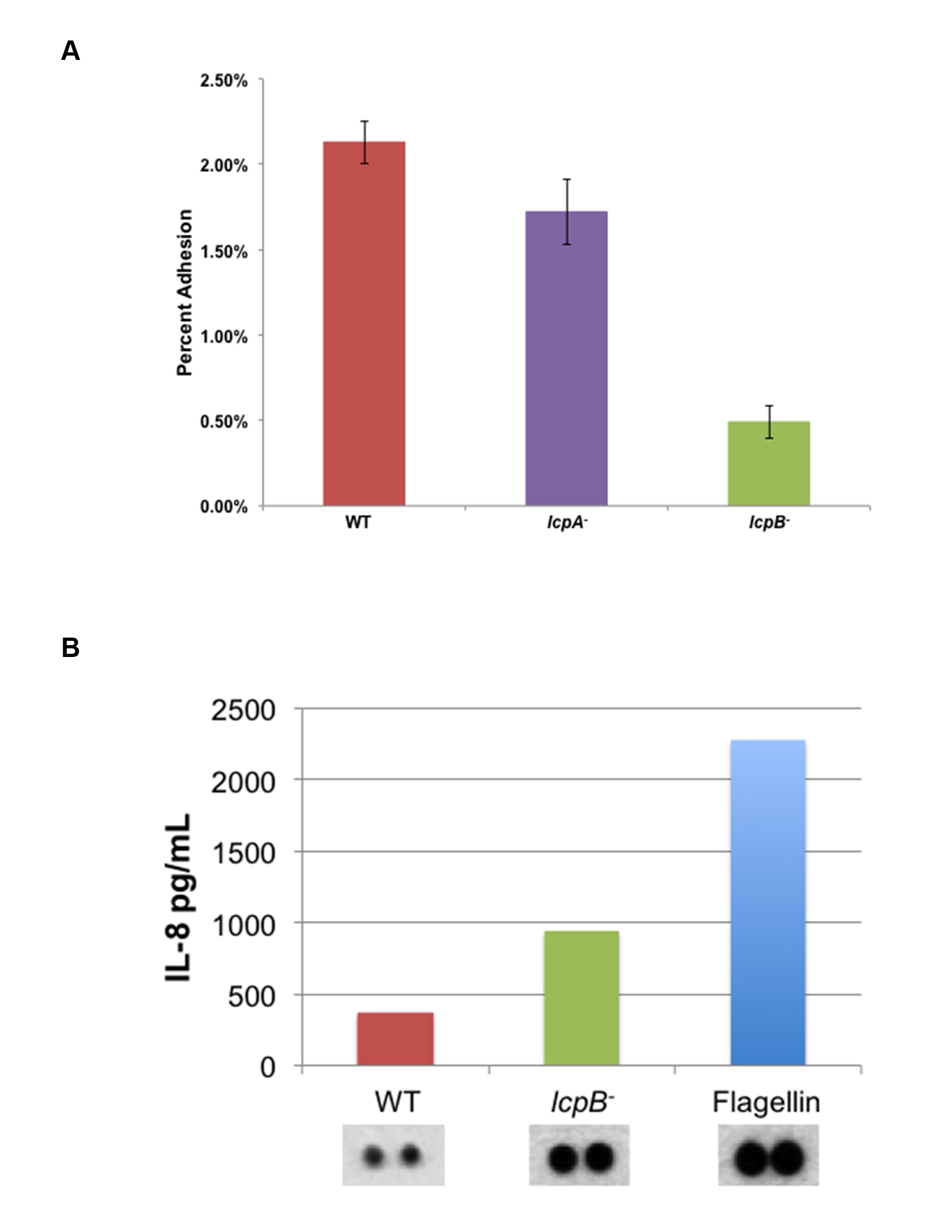

Supplement: S9 Fig — (A) Adhesion to Caco-2BBe epithelial cells was determined in the lcpA - and lcpB - mutants compared to the wild-type strain. The target MOI was 50 for the wild-type and lcpA - mutant, but 100 was used for the lcpB - mutant due to variability in percent adhesion with lower MOIs. The percent adhesion of both mutants trended less than the wild-type strain, but a Student’s t test indicate that the differences are not significant in either mutant (p > 0.05). A total of three biological replicates were done four times for each strain and error bars represent standard deviation. (B) IL-8 secretion from HT-29 epithelial cells was determined by stimulation of HT-29’s with 250μg of total protein from surface layer extracts from the lcpB - mutant and wild-type strains. Data from two different methodologies are shown (ELISA, bar graph, 1 biological replicate; and Cytokine Profiler Array, immunoblot, 1 biological replicate); purified flagellin from Salmonella typhimurium is the positive control (blue bar), and all samples were normalized using no-protein control. (TIF) [file ppat.1005946.s012.tif]

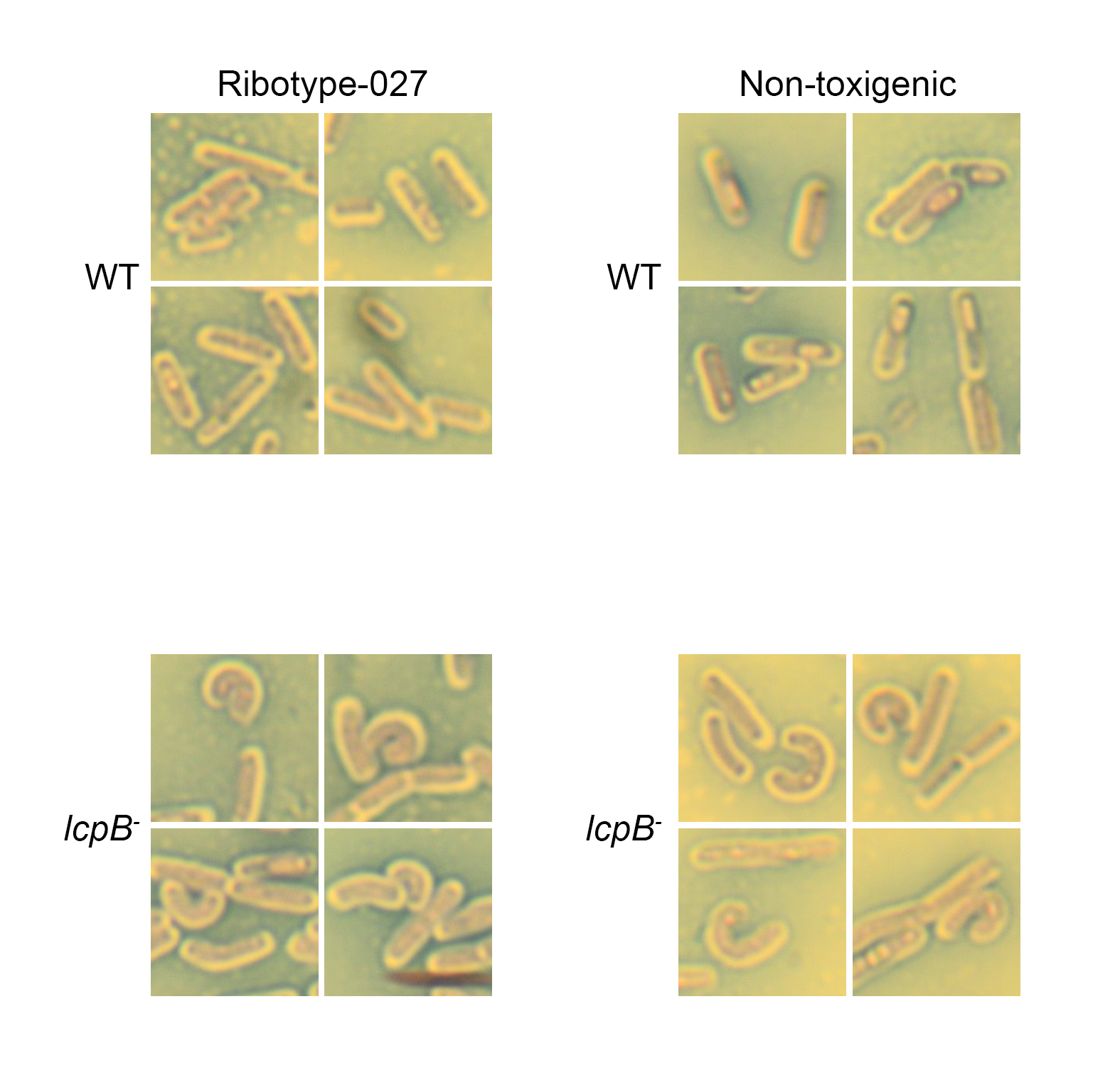

Supplement: S10 Fig — Top and bottom left panels: Parent (top; GV44; “WT”) and lcpB - (bottom; GV435) derivative, C. difficile ribotype 027 strain. Top and bottom right panels: Parent (top; GV66, “WT”) and lcpB - (bottom; GV436) derivative, non-toxigenic strain. All morphology visualized using Maneval’s capsule staining (described in Methods). (TIF) [file ppat.1005946.s013.tif]
